# Catalytic Promiscuity of cGAS: A Facile Enzymatic Synthesis of 2′‐3′‐Linked Cyclic Dinucleotides

**DOI:** 10.1002/cbic.202000433

**Published:** 2020-08-04

**Authors:** Katrin Rosenthal, Martin Becker, Jascha Rolf, Regine Siedentop, Michael Hillen, Markus Nett, Stephan Lütz

**Affiliations:** ^1^ Department of Biochemical and Chemical Engineering Chair for Bioprocess Engineering TU Dortmund University E mil-Figge-Strasse 66 44227 Dortmund Germany; ^2^ Department of Biochemical and Chemical Engineering Laboratory of Technical Biology TU Dortmund University Emil-Figge-Strasse 66 44227 Dortmund Germany

**Keywords:** biocatalysis, catalytic promiscuity, cGAS, cyclic dinucleotides, enzymes

## Abstract

Cyclic GMP‐AMP synthase (cGAS) is a cytosolic DNA sensor that catalyzes the synthesis of the cyclic GMP‐AMP dinucleotide 2′3′‐cGAMP. 2′3′‐cGAMP functions as inducer for the production of type I interferons. Derivatives of this important second messenger are highly valuable for pharmaceutical applications. However, the production of these analogues requires complex, multistep syntheses. Herein, human cGAS is shown to react with a series of unnatural nucleotides, thus leading to novel cyclic dinucleotides. Most substrate derivatives with modifications at the nucleobase, ribose, and the α‐thio phosphate were accepted. These results demonstrate the catalytic promiscuity of human cGAS and its utility for the biocatalytic synthesis of cyclic dinucleotide derivatives.

## Graphical abstract

**Enzymatic shortcut**: Cyclic dinucleotides, which are of great interest to study immunology and immune oncology, can be synthesized in a one‐step biotransformation significantly shortening the chemical synthesis route. The enzyme displays a surprisingly large substrate scope.

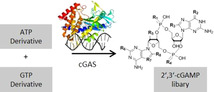

Cyclic dinucleotides are second messengers that can be found in prokaryotes and also in eukaryotes.^[1]^ Previous studies have shown that the cyclic GMP‐AMP dinucleotide 2′3′‐cGAMP is part of the innate immune system and activates the transmembrane protein stimulator of interferon genes (STING), which initiates a signal transduction cascade that produces interferons and cytokines in vertebrates.^[2]^ 2′3′‐cGAMP is produced when double‐stranded DNA is present in the cytosol.^[3]^ Cytosolic DNA serves as a danger signal and activates cyclic guanosine monophosphate (GMP)‐adenosine monophosphate (AMP) synthase (cGAS, E.C. 2.7.7.86), which catalyzes the cyclization of ATP and GTP.^[4]^ The cyclization proceeds in two steps. First, cGAS catalyzes the generation of a linear dinucleotide with a 2′‐5′ linkage.^[2a]^ Subsequently, a 3′‐5′ linkage is exclusively formed. GTP is therefore the preferred attacking nucleotide and ATP is the nucleotide being attacked.

During recent years, the structure, regulation, mechanism, and kinetics of human cGAS and other homologues have been described.[^2b^, ^4b^, ^5^] In this study, we investigate the catalytic promiscuity of human cGAS and its application as a biocatalyst. Many enzymes have been shown to efficiently catalyze promiscuous reactions.^[6]^ Substrate promiscuity provides an immediate evolutionary advantage for the biological system which is coincidently beneficial for broad synthetic applications of biocatalysts.^[7]^ Libraries of other cyclic dinucleotides than the natural 2′3′‐cGAMP are thought to be useful for getting a more detailed insight into the meaning of cyclic dinucleotides as second messenger such as STING interaction studies or the identification of unknown cyclic dinucleotide receptor proteins.^[8]^ However, larger collections of modified analogues are difficult to access.^[9]^ The chemical synthesis of cyclic dinucleotide analogues was established nearly 10 years ago.^[10]^ However, these synthetic routes suffer from low yields (<30 %) due to multiple steps, require complex protective group chemistry, and are laborious with a synthesis duration of around several days. It was recently shown that various 3′‐5′‐linked cyclic dinucleotide analogues can be synthesized by the promiscuous cyclic‐AMP‐GMP synthetase DncV from the bacteria *Vibrio cholerae*.^[11]^ However, 3′‐5′‐linked cyclic dinucleotides are not functionally active in human cells and have a low stimulatory potency for human STING.[^2a^, ^12^] Some 2′‐5′‐linked cGAMP analogues have already been synthesized with murine cGAS, such as a bisphosphothionate analogue, which is hydrolysis‐resistant with a similar affinity for the human STING receptor compared to natural 2′3′‐cGAMP.^[13]^ It is known that human cGAS also catalyzes the cyclization of GTP into c‐di‐GMP with a 2′‐5′ linkage. This is in contrast to ATP that is not able to induce the synthesis of c‐di‐AMP.[^2a^, ^5d^] However, despite many biocatalytic syntheses being applied in the pharmaceutical industry^[14]^ and despite its significant advantage of shortening a multistep procedure, currently no enzyme is applied for the industrial synthesis of cyclic dinucleotides. One reason for this might be that the catalytic capacities and potentials of cGAS as biocatalyst are not fully known and exhausted.

Herein, we show that the substrate promiscuity of human cGAS can be exploited to produce a series of 2′3′‐cGAMP analogues in a one‐step reaction superior to classical synthesis routes. The substrate derivatives were chosen with modifications at different positions in the nucleobase, ribose or α‐phosphate (Scheme 1). Each substrate derivative was tested with its natural counterpart to evaluate the individual effect of any position‐dependent variation. In total, 16 derivatives (Scheme 1) were tested in 17 reactions. As the substrates were stable in the reference reaction without enzyme, it was clear that the cyclic dinucleotides were transformed by human cGAS.

**Scheme 1:**
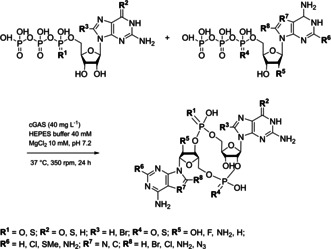
cGAS‐catalyzed conversion of substrate derivatives into cyclic dinucleotide derivatives.

The specific activities and conversions within 24 h were calculated for all reactions (Table 1). No products were synthesized for the substrates 2′d‐7‐CH‐ATP (entry 1), 2′‐NH_2_‐ATP (entry 3) and ATP‐α‐S (entry 16) and only minor amounts for the substrate 8‐Cl‐ATP (entry 13). Higher specific activities between 40 and 43 mU mg^−1^ were determined for the derivatives 8‐Br‐dATP (entry 11), 8‐Br‐ATP (entry 10) and GTP‐α‐S (entry 17) with conversions between 56 and 93 %. The specific activities are comparable to those measured for the conversion of the natural substrates ATP and GTP (74 mU mg^−1^). The highest specific activity of 136 mU mg^−1^ was measured for the product synthesis from 8‐Br‐GTP with a yield of 85 %. Thus, the addition of a bromide at the 8‐position of the nucleobase seems to be preferred for both, ATP and GTP, substrates. Another interesting substrate derivative, the α‐thio phosphate GTP analogue (entry 17), was accepted for the cyclization reaction and 93 % was converted within 24 h. This product has relevant chemical properties and is of particular importance, because the phosphothionate diester linkages are more resistant to hydrolysis than the phosphate diesters.^[13]^ For the cyclic dinucleotide resulting from 2′‐F‐ATP and GTP (entry 2), substantially higher levels of interferon production compared to the reference 2′3′‐cGAMP was recently shown using human primary blood mononuclear cell.^[15]^

**Table 1:** Enzymatic transformations of nucleotide derivatives.^[a]^

	Substrate derivative	Second substrate	Specific activity [mU mg^−1^]^[b]^	Conversion [%]^[b]^
1	2′d‐7‐CH‐ATP	GTP	0	0
2	2′‐F‐ATP	GTP	18	58^[c]^
3	2′‐NH_2_‐ATP	GTP	0	0
4	2‐Cl‐ATP	GTP	30	42
5	2‐MeS‐ATP	GTP	20	65^[c]^
6	2‐NH_2_‐PuTP	GTP	25	70^[c]^
7	2‐NH_2_‐PuTP	ATP	22	51^[c]^
8	6‐S‐GTP	ATP	20	40^[c]^
9	7‐CH‐ATP	GTP	27	88^[c]^
10	8‐Br‐ATP	GTP	43	57
11	8‐Br‐dATP	GTP	40	68
12	8‐Br‐GTP	ATP	136	85^[c]^
13	8‐Cl‐ATP	GTP	2	26
14	8‐NH_2_‐ATP	GTP	10	27^[c]^
15	8‐N_3_‐ATP	GTP	14	46
16	ATP‐α‐S	GTP	0	0
17	GTP‐α‐S	ATP	50	93^[c]^

[a] For reaction conditions and abbreviations, see the Supporting Information. [b] Measured by reversed‐phase HPLC analysis. Specific activities and conversions are based on ATP or GTP consumption. [c] Product synthesis was confirmed by ESI‐LC‐MS.

The cGAS products were additionally analyzed by electrospray ionization‐liquid chromatography‐mass spectrometry (ESI‐LC‐MS) and the expected masses were compared with the detected product masses. The synthesis of nine of the 17 products was confirmed with ESI‐LC‐MS analysis (Table 1 and the Supporting Information). Linear products or cyclic homodimers were not detected in any sample. Nevertheless, the specific activity of human cGAS was relatively low for all tested reactions, including the cyclization reaction of ATP and GTP. Using sodium phosphate buffer instead of HEPES leads to lower specific activities and conversions (see Supporting Information for details), which might be attributed to the complexation of the essential magnesium ions by the phosphate buffer.

The broad substrate promiscuity of human cGAS can be explained by the fact that most of the chosen modifications do not interact with the key residues of human cGAS. These catalytic key residues of human cGAS are Asp227 and Asp319 coordinating the amine of guanine, Lys362 coordinating the α‐phosphate of ATP, Arg376 coordinating the keto group of guanine, Ser434 coordinating the α‐phosphate of GTP, and Tyr436 stacking the adenine moiety against the aromatic residue^[4b]^ (Figure 1). It is therefore plausible that human cGAS accepts substrate derivatives with modifications at most of the chosen positions in the nucleobase, ribose or α‐phosphate. Interestingly, our measured conversions do not completely cover recently published results about the synthesis of 2′3′‐cGAMP derivatives using full‐length cGAS from human, mouse and chicken.^[16]^ Full‐length human cGAS did not catalyze the conversion of for example 2′‐F‐ATP or 6‐S‐GTP. This is in contrast to our experiments with truncated human cGAS (human cGAS without the 160 N‐terminal residues) that is obviously able to catalyze these conversions. This result indicates that truncated and full‐length cGAS might have different functionalities.

**Figure 1:**
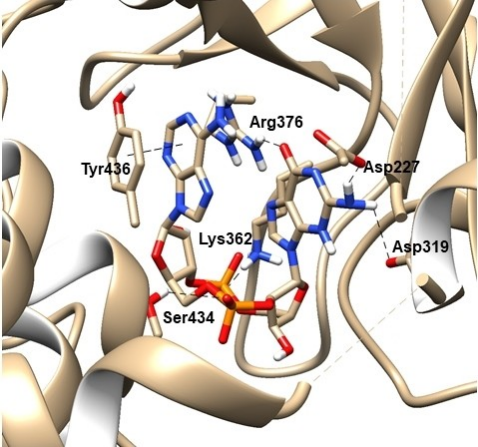
Overall structure of human cGAS in complex with 2′3′‐cGAMP. 2′3′‐cGAMP binds to human cGAS (PDB ID: 6MJX) through multiple residues. Molecular graphics and analyses performed with UCSF Chimera, developed by the Resource for Biocomputing, Visualization, and Informatics at the University of California, San Francisco, with support from NIH P41‐GM103311^[17]^.

Based on our findings mg‐scale biotransformations of 2′‐F‐ATP, 8‐Br‐ATP, 8‐Cl‐ATP, 8‐NH_2_‐ATP, and 8‐N_3_‐ATP with GTP into the corresponding cyclic dinucleotide derivative were carried out. Thus, 10 μmol of the substrates (5.8 mg 2′‐F‐ATP, 5.8 mg 8‐Br‐ATP, 5.4 mg 8‐Cl‐ATP, 5.2 mg 8‐NH_2_‐ATP, or 5.4 mg 8‐N_3_‐ATP and 5.2 mg GTP) were converted into the corresponding cyclic dinucleotide derivative using cGAS within 24 h. The conversion of 5 μmol ATP and GTP served as a reference reaction. The reactions resulted in 65 % conversion of GTP with 2′‐F‐ATP, 47 % GTP with 8‐Br‐ATP, 35 % GTP with 8‐Cl‐ATP, 55 % GTP with 8‐NH_2_‐ATP, and 58 % GTP with 8‐N_3_‐ATP. These conversions were similar, but not fully comparable with the biotransformations on analytical scale, which can only be attributed to the change in the reaction scale. HPLC Purification of 2′3′‐cGAMP yielded 1.1 mg of product. The derivatives were purified by two‐step HPLC to give 0.1 mg cyclic GMP‐2′‐F‐AMP, and 4.1 mg cyclic GMP‐8‐NH_2_‐AMP. The syntheses of cGAMP, cyclic GMP‐2′‐F‐AMP and cyclic GMP‐8‐NH_2_‐AMP were successfully validated by NMR spectroscopy (see the Supporting Information for details). The products cyclic GMP‐8‐Br‐AMP, cyclic GMP‐8‐N_3_‐AMP and cyclic GMP‐8‐Cl‐AMP were not purified in quantifiable amounts.

In summary, we have reported a short and facile enzymatic synthesis of novel unnatural cyclic dinucleotides based on the promiscuous activity of human cGAS. Remarkably, most tested ATP and GTP substrate derivatives with modifications at the nucleobases, riboses and α‐phosphate were converted into cyclic dinucleotides with their natural counterpart.

The relevance of these cyclic dinucleotide derivatives was recently demonstrated by investigating the biological activity towards the STING receptor based on the analysis of the structure‐activity relationship and mouse model systems.^[16]^ Next to the bisphosphothionate analogue, it turned out that especially cyclic dinucleotides with a fluorine substitution in the 2′‐position of the adenosine ribose shows improved STING binding.^[16]^ We were now able to synthesize cyclic GMP‐2′‐F‐AMP using a biocatalyst instead of a complex chemical route. We additionally synthesized for the first time derivatives with modifications at the 8‐position of the nucleoside. The catalytic promiscuity and the successful application in preparative scale‐synthesis elucidates the potential of cGAS as a viable catalyst for cyclic dinucleotide synthesis. Moreover, the vast biological diversity of cGAS holds the promise for more applicable biocatalysts.^[5i]^

## Experimental Section

**Analytical scale biotransformation**: For enzyme assays, 40 mM HEPES, 10 mM MgCl_2_, pH 7.2 was used with 0.1 mg mL^−1^ Herring testis DNA and 40 μg mL^−1^ human cGAS. The reaction was started by adding 0.5 mM substrate derivative and 0.5 mM substrate, either ATP or GTP. The reaction volume was 1 mL. Negative controls were performed analogously without the addition of enzyme. All samples were incubated at 37 °C and 300 rpm in an orbital shaker for at least 24 h. Reactions were stopped by heating at 95 °C for 5 min. The samples were analyzed with HPLC and LC‐MS. The activities given in Table 1 are the result of following the enzyme activity for 24 h and determining the slope of substrate consumption for the initial 4 to 5 h.

**Milligram‐scale biotransformation**: For milligram‐scale biotransformation, 20 mL reaction solution (40 mM HEPES, 10 mM MgCl_2_, pH 7.2 with 0.1 mg mL^−1^ Herring testis DNA, 0.5 mM substrate derivative and 0.5 mM GTP) was used in shaking flasks. The reaction was started by adding 40 μg mL^−1^ human cGAS. The reaction mixture was incubated at 37 °C and 200 rpm in an orbital shaker for 24 h. The reaction was stopped by heating at 95 °C for 5 min. The product solutions were frozen and stored at −20 °C.

**Fractionation of biotransformation products by HPLC**: The frozen samples were lyophilized to dryness, subsequently resolved in 1 mL H_2_O and centrifuged at 21 000 g for 5 min. The supernatant of the product solution was separated and collected as fractions with a LaChrome Elite HPLC system (VWR, Darmstadt, Germany) equipped with an ISAspher 100‐3C18AQ column, 150×3 mm (Isera GmbH, Düren, Germany). The column temperature was set to 30 °C. The flow rate was set to 1 mL min^−1^. The product solution was repetitively injected with volumes of 99.5 μL. A gradient of 50 mM triethylamine acetate (TEAA) with 3 % *v*/*v* acetonitrile (solvent A) and 100 % acetonitrile (solvent B) was used for the chromatography. The solvent gradient for all products was: 0–10 min: 0 % B, 10–20 min: 0 to 30 % B, 20–22 min: 30 % B, 22–25 min 30 to 0 % B, 25–30 min 0 % B. Fractions were taken: cGAMP: 12–13 min; cyclic GMP‐2′‐F‐AMP: 11–12 min and 17–18 min; cyclic GMP‐8‐Br‐AMP: 17–19 min; cyclic GMP‐8‐Cl‐AMP: 16–18 min; cyclic GMP‐8‐NH_2_‐AMP: 11–13 min; cyclic GMP‐8‐N_3_‐AMP: 16–17 min. Elution of compounds was monitored with a UV detector at 254 nm. The collected fractions were lyophilized and product purification was validated with NMR analysis.

For further purification of the product derivatives, a second fractionation using chromatography was performed. In the second fractionation, the solvent gradient was individually adapted for each compound: cyclic GMP‐2′‐F‐AMP: 0–20 min: 0 to 22.5 % B, 20–22 min: 22.5 to 90 % B, 22–30 min: 90 % B, 30–32 min 90 to 0 % B, 32–42 min 0 % B; Fractions were taken: 8.7–10.2 min; cyclic GMP‐8‐Br‐AMP: 0–20 min: 0 to 15 % B, 20–22 min: 15 to 90 % B, 22–30 min: 90 % B, 30–32 min: 90 to 0 % B, 32–42 min: 0 % B; Fractions were taken: 14.3–16.3 min; cyclic GMP‐8‐Cl‐AMP: 0–5 min: 0 % B, 5–25 min: 0 to 30 % B, 25–27 min: 30 to 90 % B, 27–35 min: 90 % B, 35–37 min: 90 to 0 % B, 37–47: 0 % B; Fractions were taken:12.7–14.1 min and 16.3–18.3 min; cyclic GMP‐8‐NH_2_‐AMP: 0–18 min: 0 % B, 18–28 min: 0 to 30 % B, 28–30 min: 30 to 90 % B, 30–38 min: 90 % B, 38–40 min: 90 to 0 % B, 40–50 min: 0 % B; Fractions were taken: 25.9–27.9 min; cyclic GMP‐8‐N_3_‐AMP: 0–15 min: 0 to 22.5 % B, 15–17 min: 22.5 to 90 % B, 17–25 min: 90 % B, 25–27 min: 90 to 0 % B, 27–37 min: 0 % B; Fractions were taken: 7.1–9.1 min. Elution of compounds was monitored with a UV detector at 254 nm. The collected fractions were lyophilized and product purification was validated with NMR analysis.

**NMR analysis**: NMR spectra were recorded on a Bruker AV 600 Avance III HD system (Bruker BioSpin GmbH, Rheinstetten, Germany) with D_2_O as solvent and 25 μL [D_4_]methanol as internal standard. The solvent signals were referenced to *δ*_H_ 3.31 ppm and *δ*_C_ 49.0 ppm.

*Cyclic GMP‐8‐NH_2_‐AMP*: ^1^H NMR (600 MHz, D_2_O/CD_3_OD): *δ*=8.08 (s, 1H; CH), 7.94 (s, 1H; CH), 6.17 (s, 1H; CH), 5.99 (d, *J*=8.2 Hz, 1H; CH), 5.60 (dt, ^3^ *J*_H,H_=8.6 Hz, 4.3 Hz, 1H; CH), 5.06 (d, *J*=7.4 Hz, 1H; CH), 4.72 (d, *J*=5.'6 Hz, 1H; CH), 4.62 (d, *J*=4.3 Hz, 1H; CH), 4.44 (d, *J*=2.4 Hz, 1H; CH), 4.38 (s, 1H; CH), 4.27 (m, 2H; CH_2_), 4.37, 4.14 ppm (m, 2H; CH_2_); ^13^C NMR (600 MHz, D_2_O/CD_3_OD): *δ*=160.2 (CCO), 154.5 (CN), 153.2 (CN), 153.1 (CN), 150.1 (CH), 149.7 (CN), 142.3 (CH), 118.1 (CN), 117.1 (CN), 91.5 (CH), 86.8 (CH), 84.6 (CH), 80.6 (CH), 75.6 (CH), 74.7 (CH), 72.4 (CH), 71.7 (CH), 66.7 (CH_2_), 62.9 ppm (CH_2_).

*Cyclic GMP‐8‐F’‐AMP*: ^1^H NMR (600 MHz, D_2_O/CD_3_OD): *δ*=8.30 (s, 1H; CH), 8.29 (s, 1H; CH), 7.87 (s, 1H; CH), 6.47 (m, 1H; CH), 5.96 (m, 1H; CH), 5.67 (dt, ^3^ *J*_H,H_=8.2 Hz, 4.3 Hz, 1H; CH), 5.56 (m, 1H; CH), 5.12 (m, 1H; CH), 4.60 (d, *J*=4.3 Hz, 1H; CH), 4.57 (m, 1H; CH), 4.40 (m, 1H; CH), 4.25 (m, 2H; CH_2_), 4.16 ppm (m, 2H; CH_2_); ^13^C NMR (600 MHz, D_2_O/CD_3_OD): *δ*=n.d. (CCO), n.d. (CN), 156.0 (CN), 155.1 (CH), 152.2 (CN), 148.1 (CN), 142.9 (CH), 140.7 (CH), 119.2 (CN), 118.1 (CN), 87.8 (CH), 87.6 (CH), 87.2 (CH), 84.0 (CH), 74.6 (CH), 72.2 (CH), 71.8 (CH) 69.9 (CH), n.d. (CH), 66.8 ppm (CH). (n.d.=not detected)

## Conflict of interest

The authors declare no conflict of interest.

## Supporting information

As a service to our authors and readers, this journal provides supporting information supplied by the authors. Such materials are peer reviewed and may be re‐organized for online delivery, but are not copy‐edited or typeset. Technical support issues arising from supporting information (other than missing files) should be addressed to the authors.

Supplementary
