# Supplementary material for: Catalytic Promiscuity of cGAS: A Facile Enzymatic Synthesis of 2′‐3′‐Linked Cyclic Dinucleotides
Source: Chembiochem. 2020 Aug 4;21(22):3225–8. doi: 10.1002/cbic.202000433 (PMC7754487; doi:10.1002/cbic.202000433)
Supplement: Supplementary file 1 — Supplementary [file CBIC-21-3225-s001.pdf]

ChemBioChem

Supporting Information

Catalytic Promiscuity of cGAS: A Facile Enzymatic Synthesis of 2′-3′-Linked Cyclic Dinucleotides

Katrin Rosenthal, Martin Becker, Jascha Rolf, Regine Siedentop, Michael Hillen, Markus Nett, and Stephan Lütz*

Supporting information

Materials and Methods

Substrates and reference substances were purchased in the highest purity available from BIOLOG Life Science Institute, Forschungslabor und Biochemica-Vertrieb GmbH, Germany (2‘-F-ATP, 8-Cl-ATP, 2’-NH_2-ATP, 8-NH_2-ATP, 2‘d-7-CH-ATP, 8-N_3-ATP, 2-Cl-ATP, ATP-α-S, 2-MeS-ATP, 2-NH_2-PuTP, 7-CH-ATP, 6-T-GTP, 8-Br-ATP, 8-Br-GTP, 8-Br-dATP, GTP-α-S), Sigma-Aldrich (ATP, GTP) or Invivogen (2’3’-cGAMP). The list of chemical molecules can be found in Figure S1 and the list of abbreviations can be found in Table S1. The used restriction enzymes were purchased from New England Biolabs, USA. The T4 DNA ligase, DNA ladder (GeneRuler 1 kb Plus DNA Ladder) and protein ladder (PageRuler™ Unstained Protein Ladder) were purchased from Thermo Fisher Scientific, Germany.

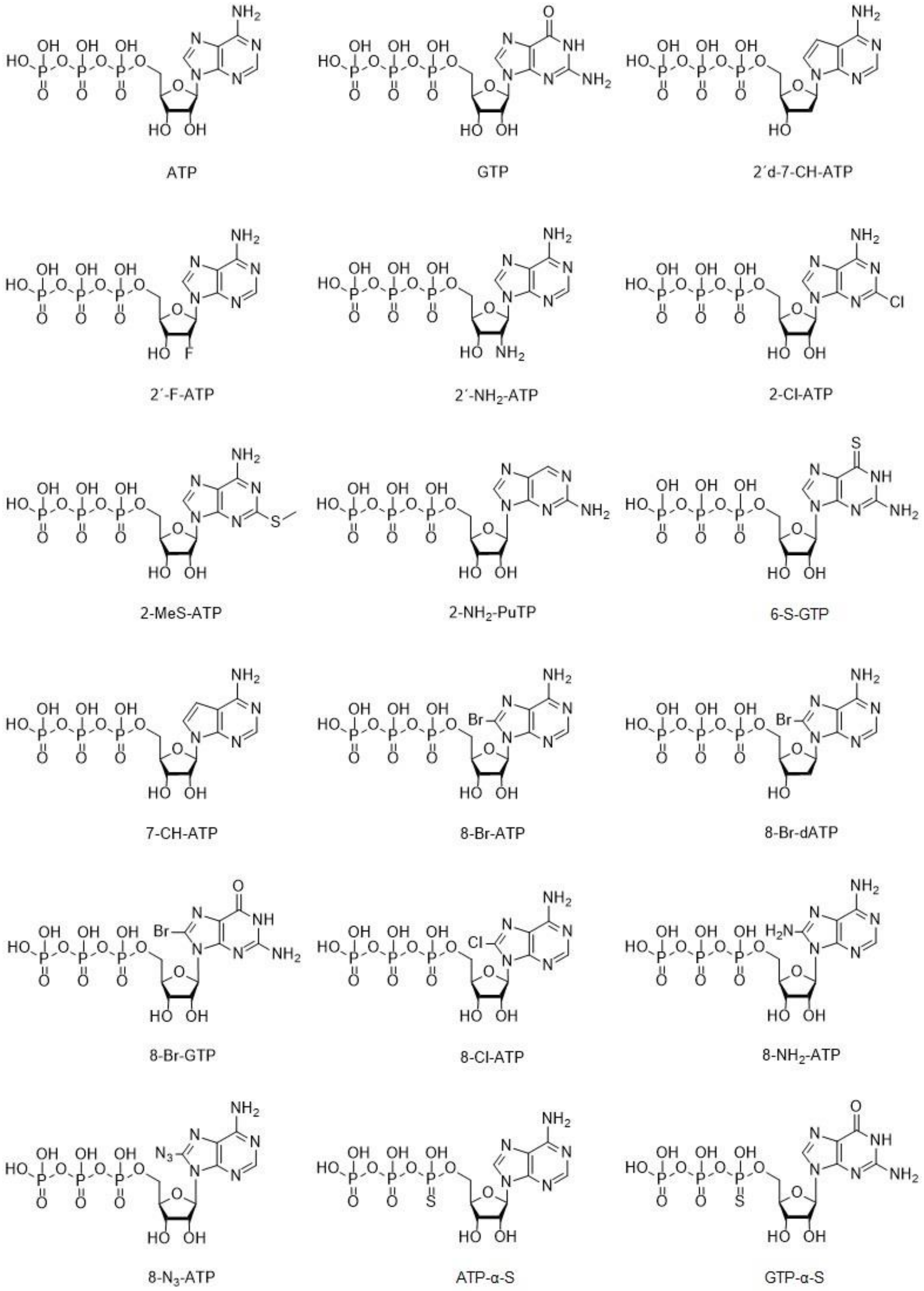

Figure S1. ATP, GTP and derivatives used as substrates for cyclic dinucleotide synthesis with cGAS as biocatalyst. List of chemical molecule abbreviations can be found in Table S1.

Table S1. List of chemical molecule abbreviations for the used substrate derivatives.

Substrates	
2‘d-7-CH-ATP	7- Deaza-2'-deoxyadenosine-5'-O-triphosphate
2‘-F-ATP	2'-Deoxy-2'-fluoroadenosine-5'-O-triphosphate
2’-NH_2-ATP	2'-Amino-2'-deoxyadenosine- 5'-O-triphosphate
2-Cl-ATP	2-Chloroadenosine-5'-triphosphate
2-MeS-ATP	2-Methylthioadenosine-5'-O-triphosphate
2-NH_2-PuTP	2-Aminopurine riboside-5'-O-triphosphate
6-S-GTP	6-Thioguanosine-5'-O-triphosphate
7-CH-ATP	7-Deazaadenosine-5'-O-triphosphate
8-Br-ATP	8-Bromoadenosine- 5'-O-triphosphate
8-Br-dATP	8-Bromo-2'-deoxyadenosine-5'-O-triphosphate
8-Br-GTP	8-Bromoguanosine-5'-O-triphosphate
8-Cl-ATP	8-Chloroadenosine-5'-triphosphate
8-NH_2-ATP	8-Aminoadenosine-5'-O-triphosphate
8-N_3-ATP	8-Azidoadenosine- 5'-O-triphosphate
ATP-α-S	Adenosine-5'-O-(1-thiotriphosphate), Rp- isomer
GTP-α-S	Guanosine-5'-O-(1-thiotriphosphate), Rp- isomer

Cloning, expression, and purification

The plasmid pET-28a(+) SUMOthscGAS encoding the human cGAS with an N-terminal His_6-tag and N-terminal SUMO-tag was used for expression (Figure S2). The genes coding for human cGAS and SUMO were purchased from Eurofins, Germany, and amplified by PCR. The purified PCR products were restricted with HindIII and NdeI and ligated into the expression vector pET-28a(+). The plasmid construct was verified by sequencing (Seqlab, Germany).

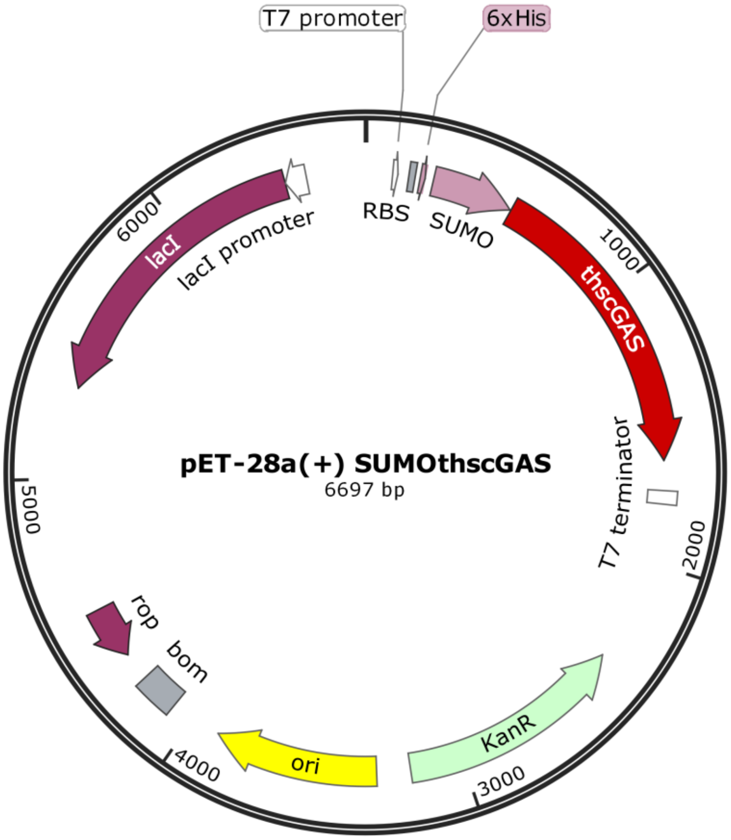

Figure S2. Plasmid map of pET-28a(+) SUMOthscGAS used for the expression of genes encoding cGAS.

Electrocompetent *E. coli* BL21 (DE3) pLysS cells were transformed with the verified plasmid for expression. The cells were grown in 200 mL 2xYT medium supplemented with 20 µg mL^{-1} chloramphenicol and 50 µg mL^{-1} kanamycin at 37 °C. As soon as the OD_{600} reached 1, cells were cooled on ice for 15 min. The expression was induced by addition of isopropyl-β-D-1-thiogalactopyranoside with a final concentration of 0.5 mM. The culture was shaken for 11 h at 20 °C and 200 rpm in an orbital shaker. The cells were harvested by centrifugation (25 min, 4 °C, 4,700 rpm) in aliquots of approximately 40 mg_{CDW}. The cell pellet was resuspended in lysis buffer (40 mM Tris-HCl, 300 mM NaCl, 40 mM imidazole, 1 mM TCEP, pH 8.0) and lysed by sonication (Branson Digital Sonifier, Branson, USA) (5 × 30 s, duty cycle 50 %, amplitude 10 %). The soluble protein was purified by Ni^{2+}-affinity chromatography and by desalting with a PD-10 column containing Sephadex G-25. The protein

concentration was determined by Bradford assay and analyzed by SDS-PAGE (Figure S3). The protein was purified with a yield of about 21 mg g_{CDW}^{-1}.

HPLC analysis

Samples were analyzed by HPLC based on a previously described method [1]. An AZURA® HPLC (KNAUER Wissenschaftliche Geräte GmbH, Berlin, Germany) was used which was equipped with the following components: AZURA® P 6.1L Isocratic Pump, AZURA® autosampler AS 6.1L, a column thermostat AZURA® CT 2.1 and a AZURA® multiwavelength detector MWD 2.1L and an ISAspher 100-3 C18AQ column (150 x 3 mm). The mobile phase A consisted of 60 mM K_2HPO_4 and 40 mM KH_2PO_4 (pH 7.0) and mobile phase B consisted of 95 % ACN. The elution program was as follows: 0 min 100 % A, 0 % B; 3 min 100 % A, 0 % B; 5 min 95 % A, 5 % B; 7 min 80 % A, 20 % B; 8.5 min 75 % A, 25 % B; 12 min 60 % A, 40 % B; 15 min 100 % A, 0 % B; 20 min 100 % A, 0 % B. The flow rate was 1 mL min^{-1} and the column was heated to 35 °C. Peaks were detected at 254 nm. The injection volume was 5 µL.

LC-MS analysis

Samples were analyzed by LC-MS using an Agilent Technologies (Santa Clara, USA) 1260 Infinity II LC system equipped with Diode Array Detector (1260 DAD HS), Multicolumn Thermostat (1260 MCT), Degasser (1260 Degasser), Multisampler (1260 Multisampler), Binary Pump (1260 Binary Pump) and an Agilent Poroshell 120 EC-C18 (4.6 x 100 mm, 2.7 µm particle size) column. The mobile phase A consisted of 0.1 % formic acid and mobile phase B consisted of 100 % ACN. The elution program was as follows: 0 min 5 % A, 95 % B; 7 min 95 % A, 5 % B; 9 min 5 % A, 95 % B; 14 min 5 % A, 95 % B. The flow rate was 1 mL min^{-1} and the column was heated to 40 °C. Peaks were detected at 254 nm. The injection volume was 5 µL. The mass spectra were obtained with an Agilent Technologies 6120 Single Quadrupole LC-MS between a mass range from m/z 100 to 1000. In the electrospray chamber a temperature of 350 °C was set and a dry gas flow rate of 12 L min^{-1}. The nebulizer pressure was 35 psi and the capillary voltage 3 kV.

SDS gel

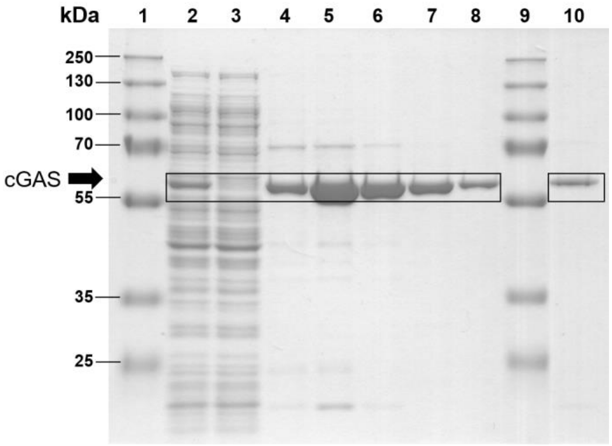

Figure S3. SDS-PAGE analysis of purified proteins. 1: Ladder (Thermo Scientific PageRuler^{TM} Unstained Protein Ladder), 2: Cell lysate after centrifugation and sterile filtration, 3: IMAC flow-through fraction, 4: IMAC elution fraction 1, 5: IMAC elution fraction 2, 6: IMAC elution fraction 3, 7: IMAC elution fraction 4, 8: IMAC elution fraction 5, 9: Ladder, 10: Purified protein after buffer exchange.

DNA sequence

The sequence starts and ends with the restriction sites for NdeI and HindIII (in capital letters). The coding sequence for SUMO (NM_001180818.1) and human cGAS (NM_138441.2) is underlined.

CATATGtcggactcagaagtcaatcaagaagctaagccagaggtcaagccagaagtcaagcctgagactcacatcaatttaaaggtgtccga
tggatcttcagagatcttcttcaagatcaaaaagaccactcctttaagaaggctgatggaagcgttcgctaaaagacagggtaaggaaatggact
ccttaagattcttgtacgacggtattagaattcaagctgatcagacccctgaagatttggacatggaggataacgatattattgaggctcacagag
aacagattggtggtatgggggcctcgaagctccgggcggttttggagaagttgaagctcagccgcgatgatatctccacggcggcggggatggt
gaaaggggttgtggaccacctgctgctcagactgaagtgcgactccgcgttcagaggcgtcgggctgctgaacaccgggagctactatgagcac
gtgaagatttctgcacctaatgaatttgatgtcatgtttaaactggaagtccccagaattcaactagaagaatattccaacactcgtgcatattact
ttgtgaaatttaaaagaaatccgaaagaaaatcctctgagtcagtttttagaaggtgaaatattatcagcttctaagatgctgtcaaagtttagga
aaatcattaaggaagaaattaacgacattaaagatacagatgtcatcatgaagaggaaaagaggagggagccctgctgtaacacttcttattag
tgaaaaaatatctgtggatataaccctggctttggaatcaaaaagtagctggcctgctagcacccaagaaggcctgcgcattcaaaactggcttt
cagcaaaagttaggaagcaactacgactaaagccattttaccttgtacccaagcatgcaaaggaaggaaatggtttccaagaagaaacatggc
ggctatccttctctcacatcgaaaaggaaattttgaacaatcatggaaaatctaaaacgtgctgtgaaaacaaagaagagaaatgttgcaggaa
agattgtttaaaactaatgaaatacctttttagaacagctgaaagaaaggtttaaagacaaaaaacatctggataaattctcttcttatcatgtgaa

aactgccttctttcacgtatgtacccagaaccctcaagacagtcagtgggaccgcaaagacctgggcctctgctttgataactgcgtgacatacttt

cttcagtgcctcaggacagaaaaacttgagaattattttattcctgaattcaatctattctctagcaacttaattgacaaaagaagtaaggaatttc

tgacaaagcaaattgaatatgaaagaaacaatgagtttccagtttttgatgaattttgataaAAGCTT

Amino acid sequence

Amino acid sequence of SUMO and human cGAS.

SDSEVNQEAKPEVKPEVKPETHINLKVSDGSSEIFFKIKKTTPLRRLMEAFAKRQGKEMDSLRFLYDGIRIQADQTPE

DLDMEDNDIIEAHREQIGGMGASKLRAVLEKLKLSRDDISTAAGMVKGVVDHLLLRLKCDSAFRGVGLLNTGSYYE

HVKISAPNEFDVMFKLEVPRIQLEEYSNTRAYYFVKFKRNPKENPLSQFLEGEILSASKMLSKFRKIIKEEINDIKDTDVI

MKRKRGGSPAVTLLISEKISVDITLALESKSSWPASTQEGLRIQNWLSAKVRKQLRLKPFYLVPKHAKEGNGFQEET

WRLSFSHIEKEILNNHGKSKTCCENKEEKCCRKDCLKLMKYLLEQLKERFKDKKHLDKFSSYHVKTAFFHVCTQNPQD

SQWDRKDLGLCFDNCVTYFLQCLRTEKLENYFIPEFNLFSSNLIDKRSKEFLTKQIEYERNNEFPVFDEF

Results

HPLC analysis

Table S2. HPLC retention times (n.d. – not detected).

Substrates		Retention time [min]
ATP		4.1
GTP		2.6
1	2‘d-7-CH-ATP	8.7
2	2‘-F-ATP	8.7
3	2’-NH_2-ATP	5.9
4	2-Cl-ATP	8.6
5	2-MeS-ATP	9.9
6	2-NH_2-PuTP	3.6
7	2-NH_2-PuTP	3.6
8	6-S-GTP	3.0
9	7-CH-ATP	3.7
10	8-Br-ATP	7.6
11	8-Br-dATP	n.d.
12	8-Br-GTP	n.d.
13	8-Cl-ATP	6.9
14	8-NH_2-ATP	5.2
15	8-N_3-ATP	8.6
16	ATP-α-S	8.6
17	GTP-α-S	4.2
**Products**		**Retention time [min]**
2’3’-cGAMP		9.9
1	2‘d-7-CH-ATP + GTP	n.d.
2	2‘-F-ATP + GTP	9.3
3	2’-NH_2-ATP + GTP	n.d.
4	2-Cl-ATP + GTP	9.7
5	2-MeS-ATP + GTP	10.6
6	2-NH_2-PuTP + GTP	8.7
7	2-NH_2-PuTP + ATP	9.4
8	6-S-GTP + ATP	8.9
9	7-CH-ATP + GTP	8.8
10	8-Br-ATP + GTP	10.3
11	8-Br-dATP + GTP	10.3
12	8-Br-GTP + ATP	8.9
13	8-Cl-ATP + GTP	10.2
14	8-NH_2-ATP + GTP	8.6
15	8-N_3-ATP + GTP	8.2
16	ATP-α-S + GTP	n.d.
17	GTP-α-S + ATP	9.4

LC-MS analysis

Table S3. (n.d. – not detected).

Entry	Substrate derivative	Expected product mass [g/mol]	Expected product mass [m+H]$^+$	Detected product mass [m+H]$^+$
2'3'-cGAMP		674.4	675.1	675.1
1	2'd-7-CH-ATP + GTP	657.5	658.1	n.d.
2	2'-F-ATP + GTP	676.5	677.1	677.1
3	2'-NH_2-ATP + GTP	673.5	674.1	n.d.
4	2-Cl-ATP + GTP	708.9	709.6	n.d.
5	2-MeS-ATP + GTP	720.6	721.1	721.1
6	2-NH_2-PuTP + ATP	658.5	659.1	659.1
7	2-NH_2-PuTP + GTP	674.5	675.1	675.1
8	6-S-GTP + ATP	690.6	691.1	691.1
9	7-CH-ATP + GTP	673.5	674.1	674.1
10	8-Br-ATP + GTP	753.4	754.1	n.d.
11	8-Br-dATP + GTP	737.4	738.1	n.d.
12	8-Br-GTP + ATP	753.4	754.1	754.1
13	8-Cl-ATP + GTP	708.9	709.6	n.d.
14	8-NH_2-ATP + GTP	689.5	690.1	690.1
15	8-N_3-ATP + GTP	715.5	716.1	n.d.
16	ATP-α-S + GTP	690.6	691.1	n.d.
17	GTP-α-S + ATP	690.6	691.1	691.0

Enzymatic reactions in sodium phosphate buffer

Table S4. Enzymatic transformations of nucleotide derivatives in $NaPO_4$ buffer (n.d. – not determined).

Entry	Substrate derivative	Second substrate	Specific activity [mU mg^{-1}]	Conversion [%]
1	2'd-7-CH-ATP	GTP	5	0
2	2'-F-ATP	GTP	17	50
3	2'-NH_2-ATP	GTP	19	57
4	2-Cl-ATP	GTP	34	86
5	2-MeS-ATP	GTP	21	71
6	2-NH_2-PuTP	GTP	4	7
7	2-NH_2-PuTP	ATP	18	11
8	6-S-GTP	ATP	10	9
9	7-CH-ATP	GTP	18	68
10	8-Br-ATP	GTP	6	13
11	8-Br-dATP	GTP	34	10
12	8-Br-GTP	ATP	6	20
13	8-Cl-ATP	GTP	8	11
14	8-NH_2-ATP	GTP	6	20
15	8-N_3-ATP	GTP	n.d.	6
16	ATP-α-S	GTP	n.d.	12
17	GTP-α-S	ATP	71	76

Chromatograms and mass spectra

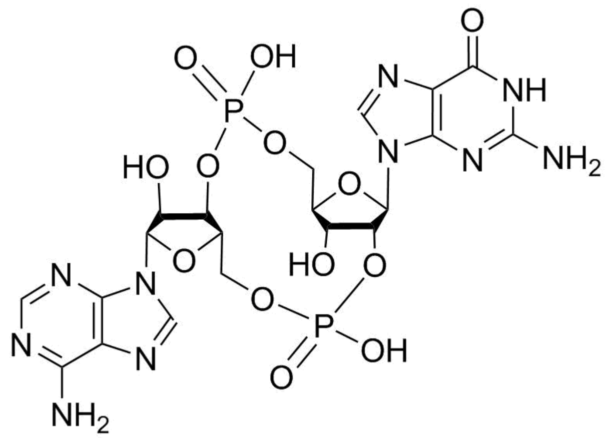

Fig. S2: Chemical structure of 2’3’-cGAMP.

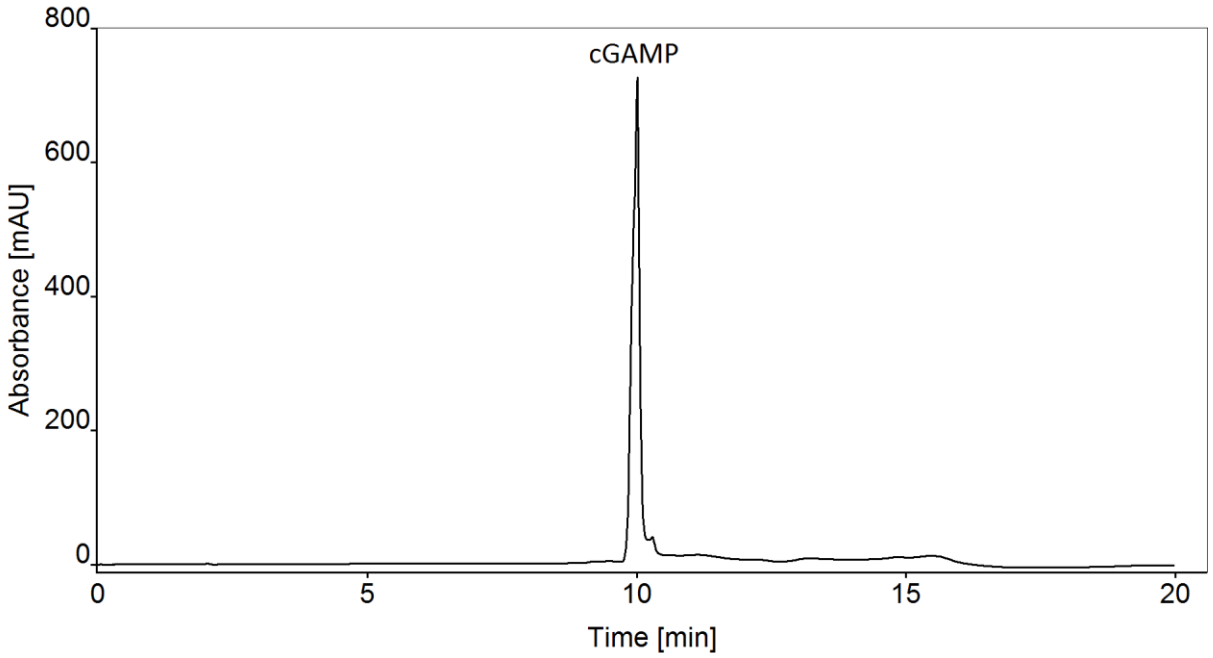

Fig. S3: Reference chromatogram of synthetic 2’3’-cGAMP (concentration 0.5 mM).

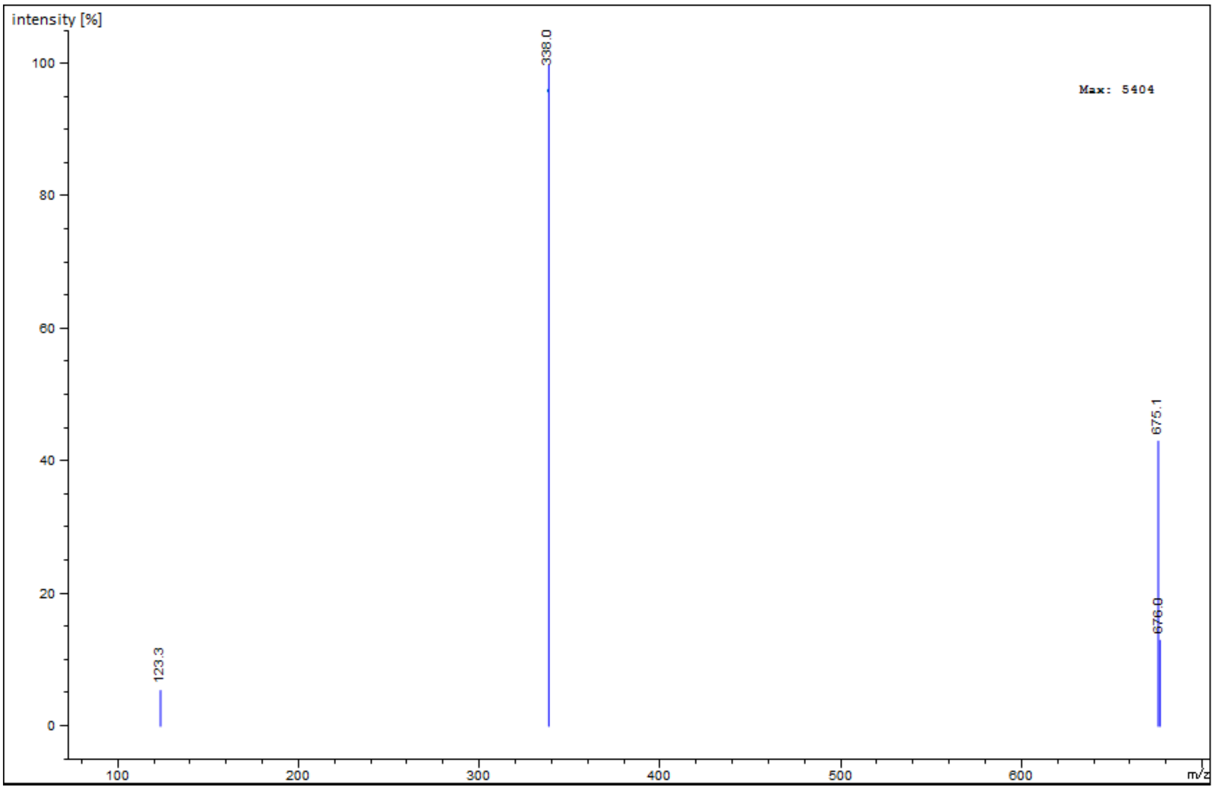

Figure S4. Reference MS spectrum of synthetic 2'3'-cGAMP ([M+H$^+$]=675.1).

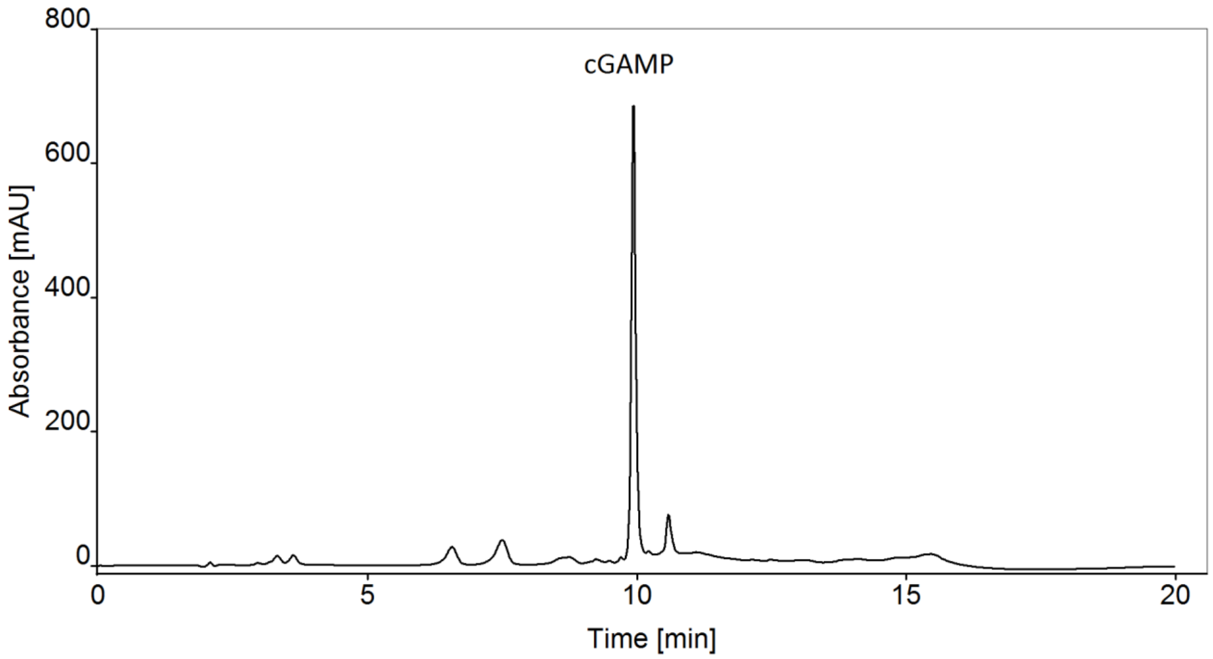

Figure S5. Chromatogram of GTP, ATP and 2’3’-cGAMP (cGAS catalyzed reaction).

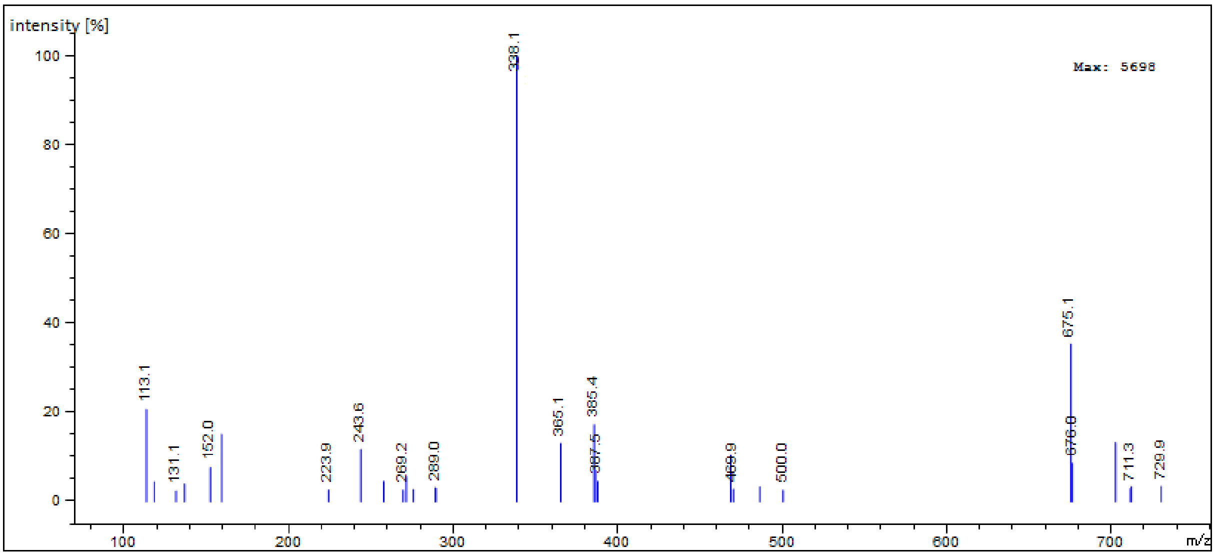

Figure S6. MS spectrum of 2’3’-cGAMP, cGAS catalyzed reaction ([M+H$^+$]=675.1).

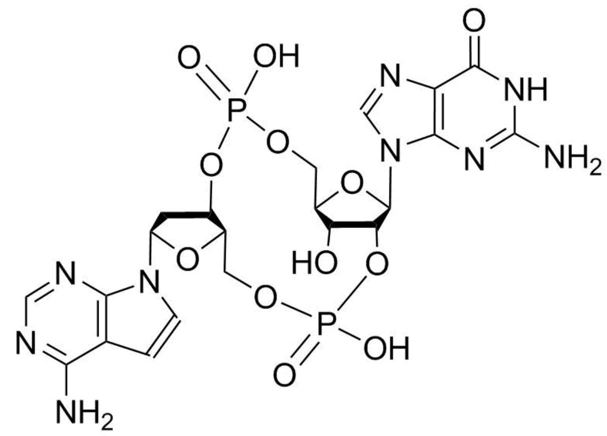

Figure S7. Chemical structure of product 1.

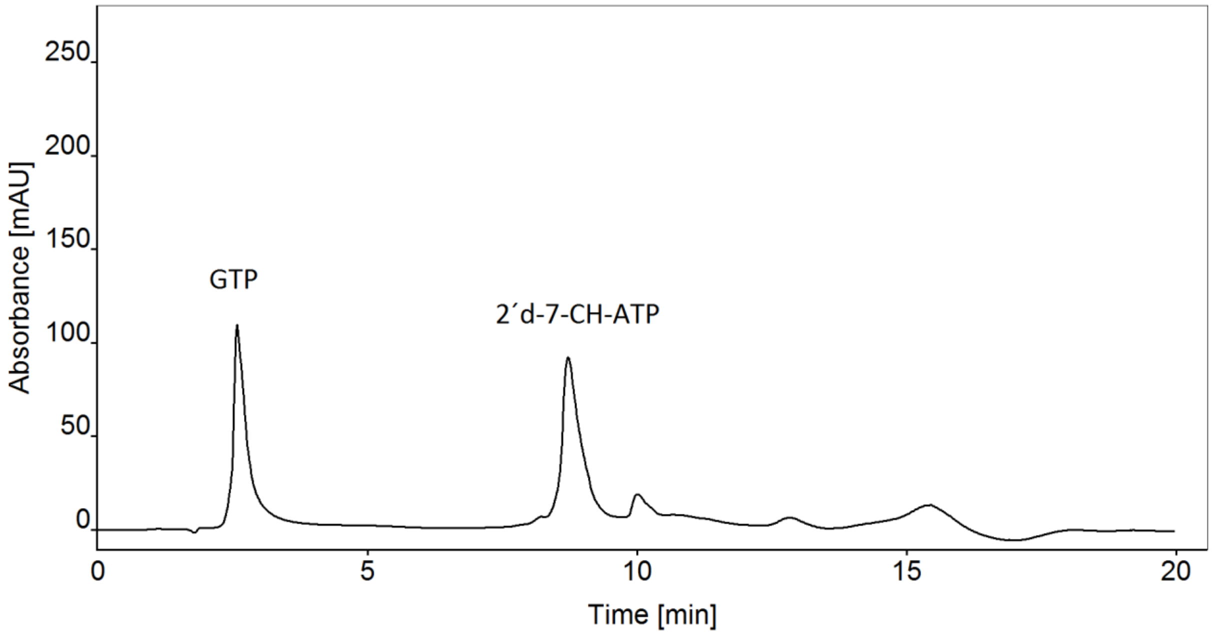

Figure S8. Chromatogram of GTP and 2´d-7-CH-ATP. Product 1 was not detected (cGAS catalyzed reaction).

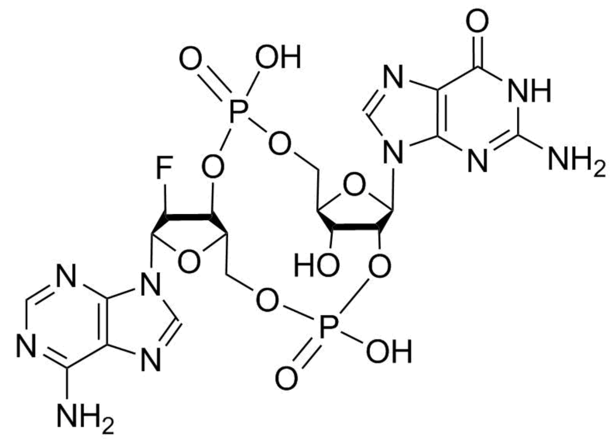

Figure S9. Chemical structure of product 2.

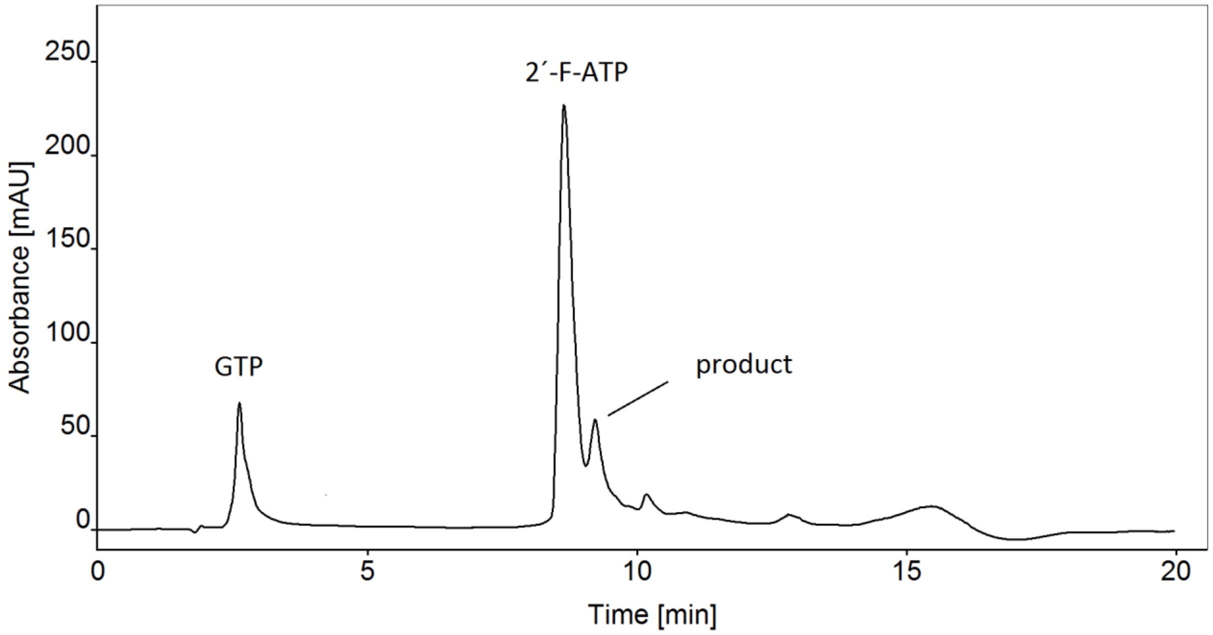

Figure S10. Chromatogram of GTP, 2´-F-ATP and product 2 (cGAS catalyzed reaction).

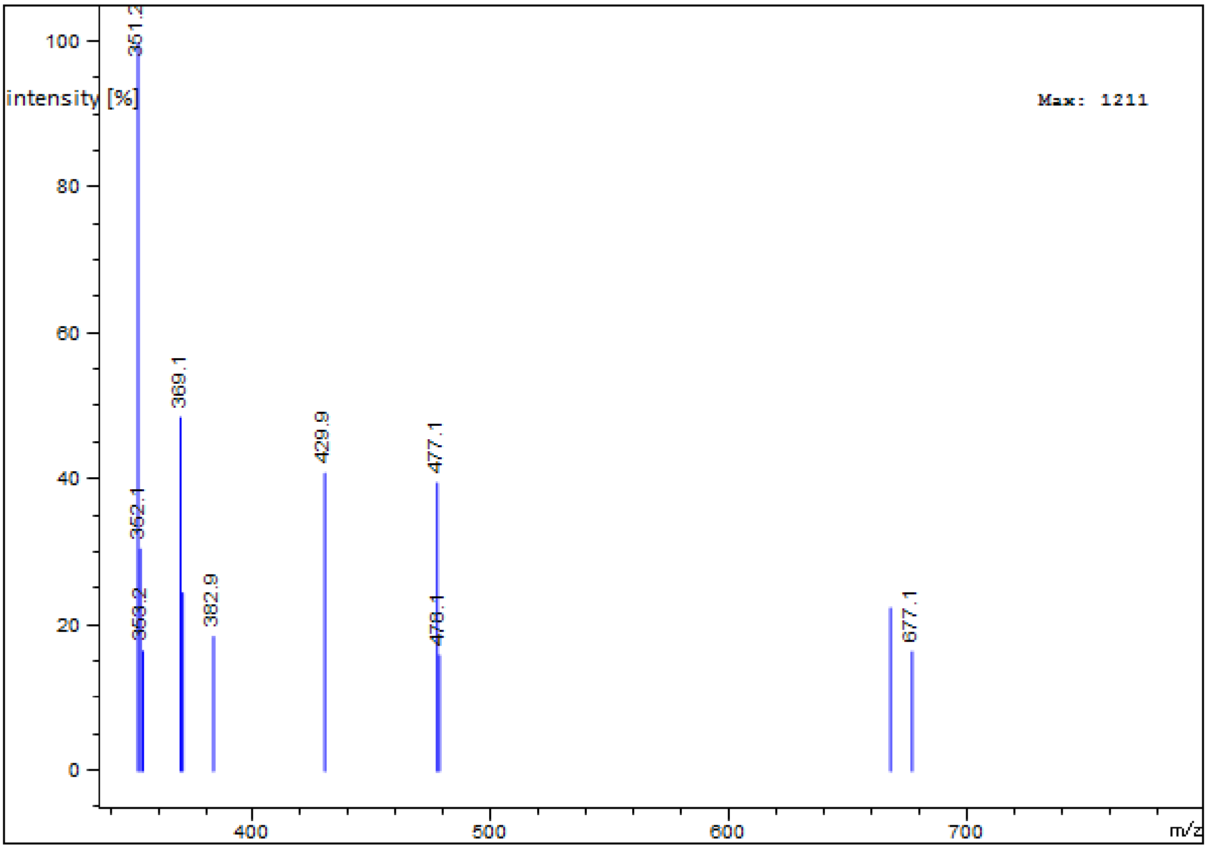

Figure S11. MS spectrum of product 2 (cGAS catalyzed reaction) ([M+H$^+$]=677.1).

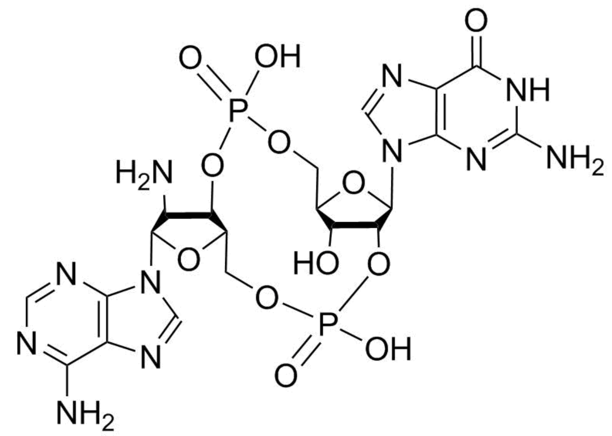

Figure S12. Chemical structure of product 3.

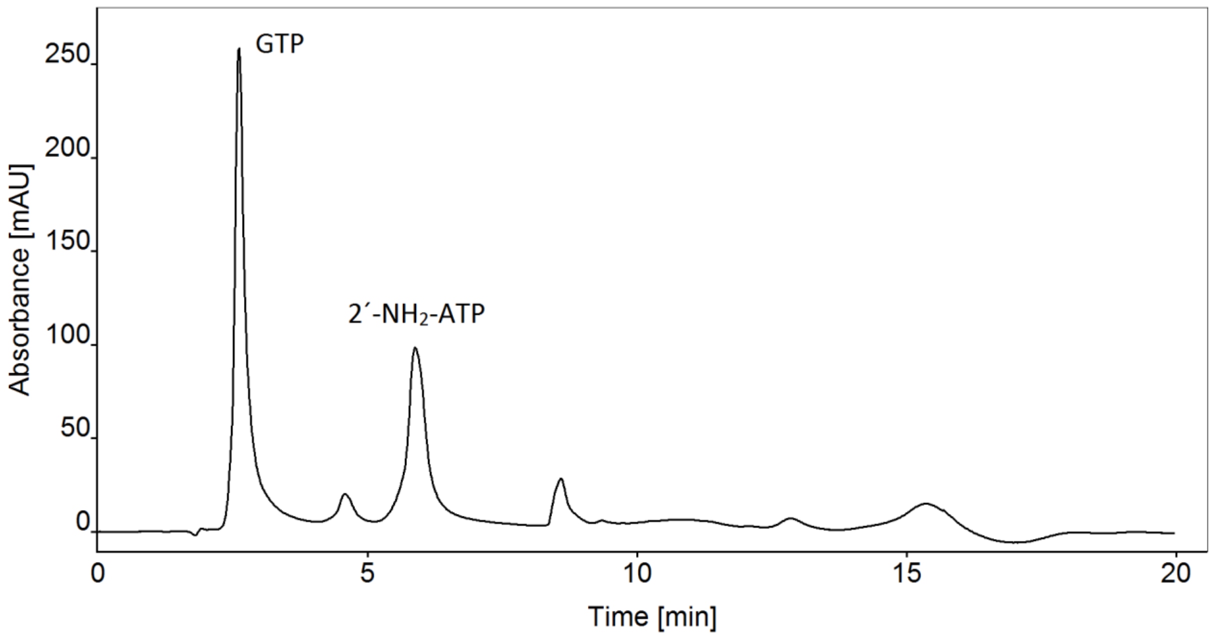

Figure S13. Chromatogram of GTP and 2´-NH_2-ATP. Product 3 was not detected (cGAS catalyzed reaction).

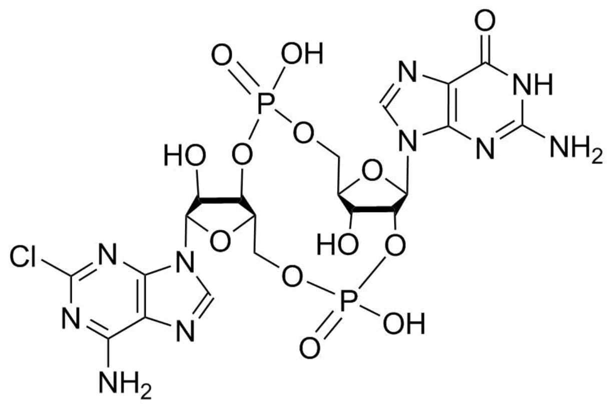

Figure S14. Chemical structure of product 4.

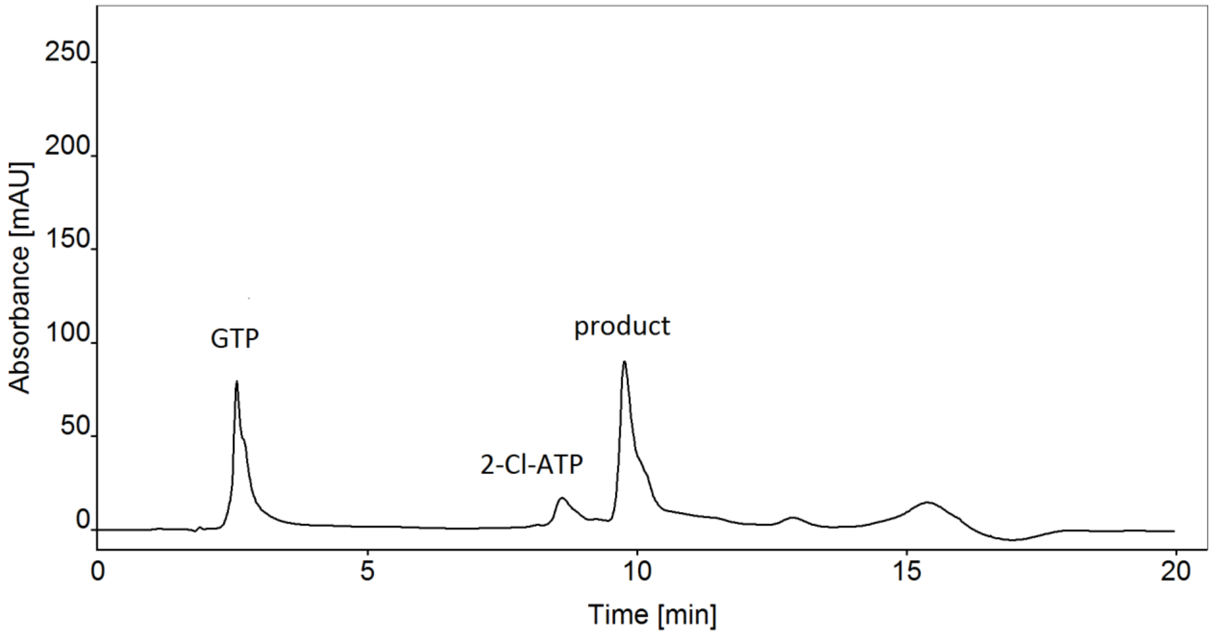

Figure S15. Chromatogram of GTP, 2-Cl-ATP and product 4 (cGAS catalyzed reaction).

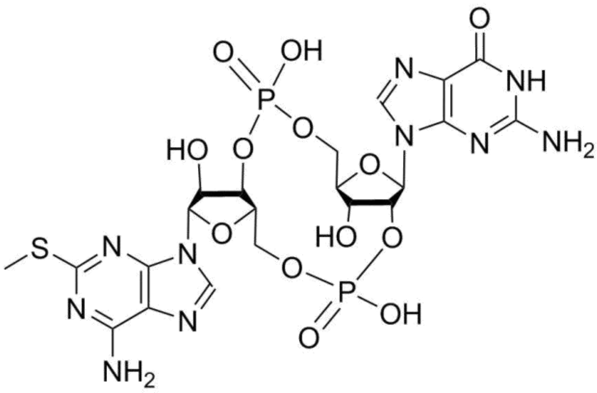

Figure S16. Chemical structure of product 5.

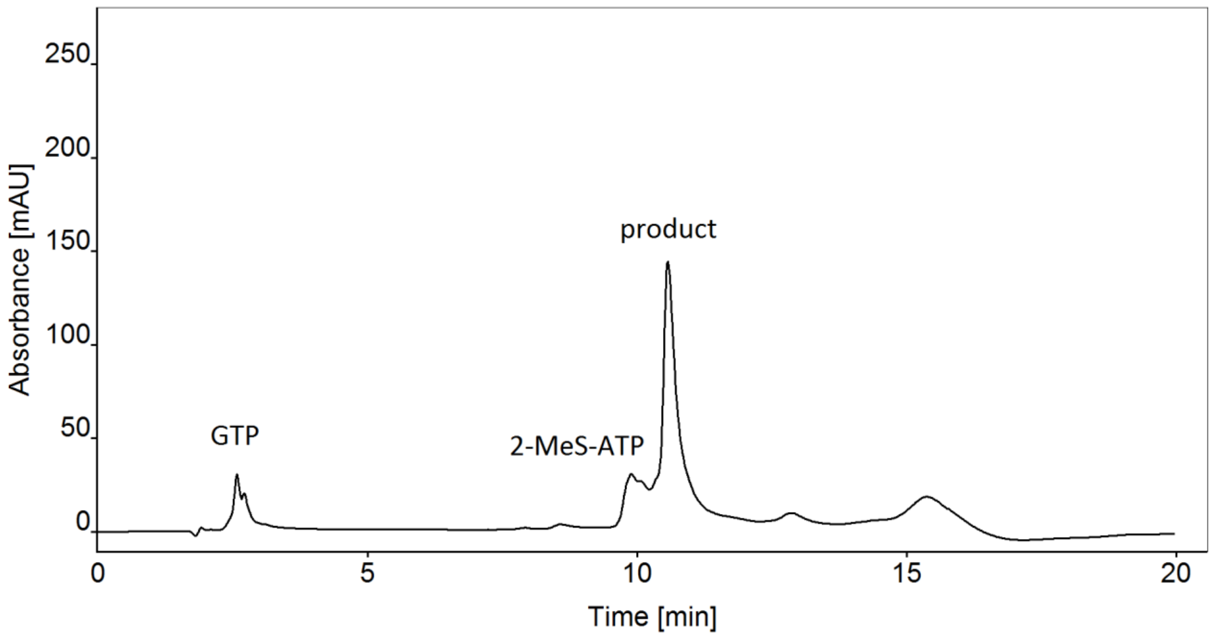

Figure S17. Chromatogram of GTP, 2-MeS-ATP and product 5 (cGAS catalyzed reaction).

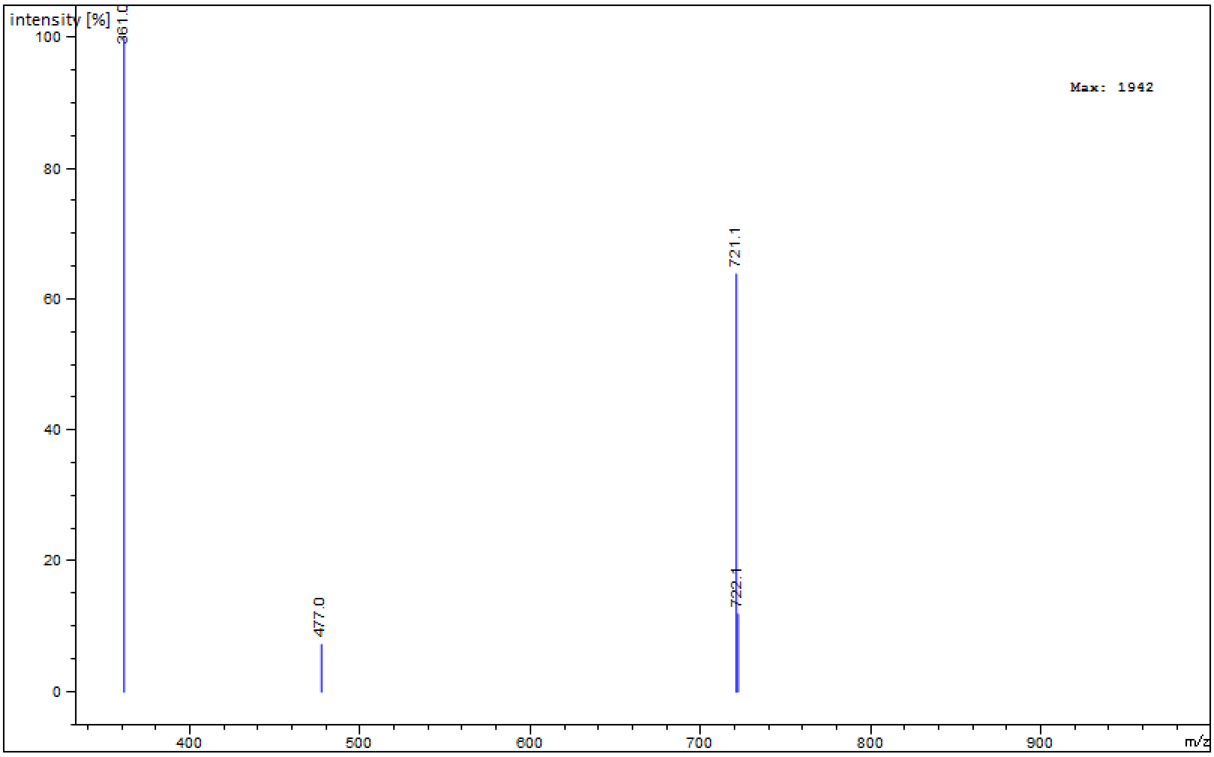

Figure S18. MS spectrum of product 5 (cGAS catalyzed reaction) ([M+H$^+$]=721.1).

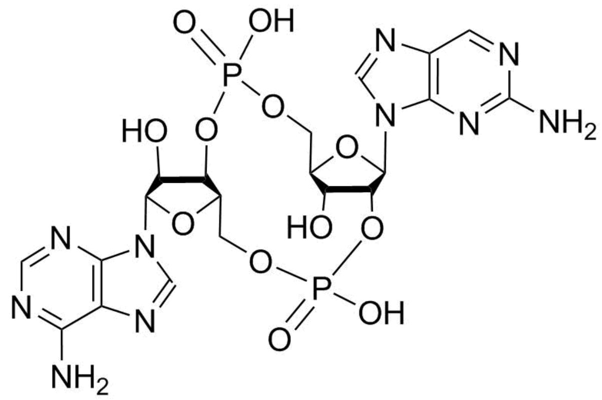

Figure S19. Chemical structure of product 6.

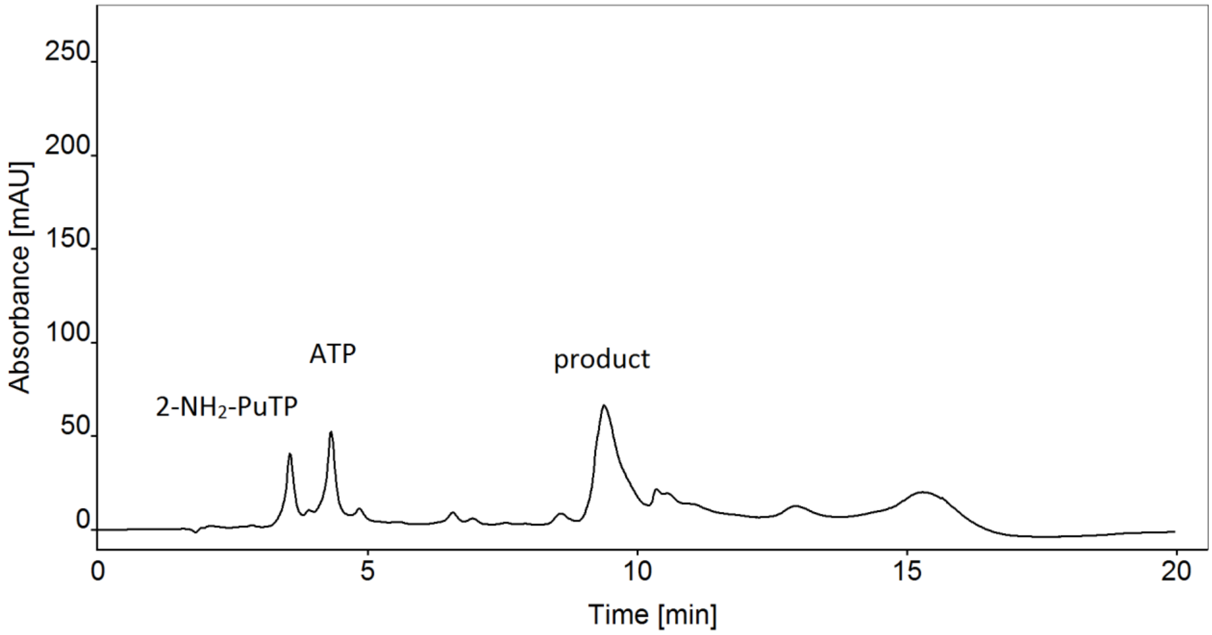

Figure S20. Chromatogram of 2-NH_2-PuTP, ATP and product 6 (cGAS catalyzed reaction).

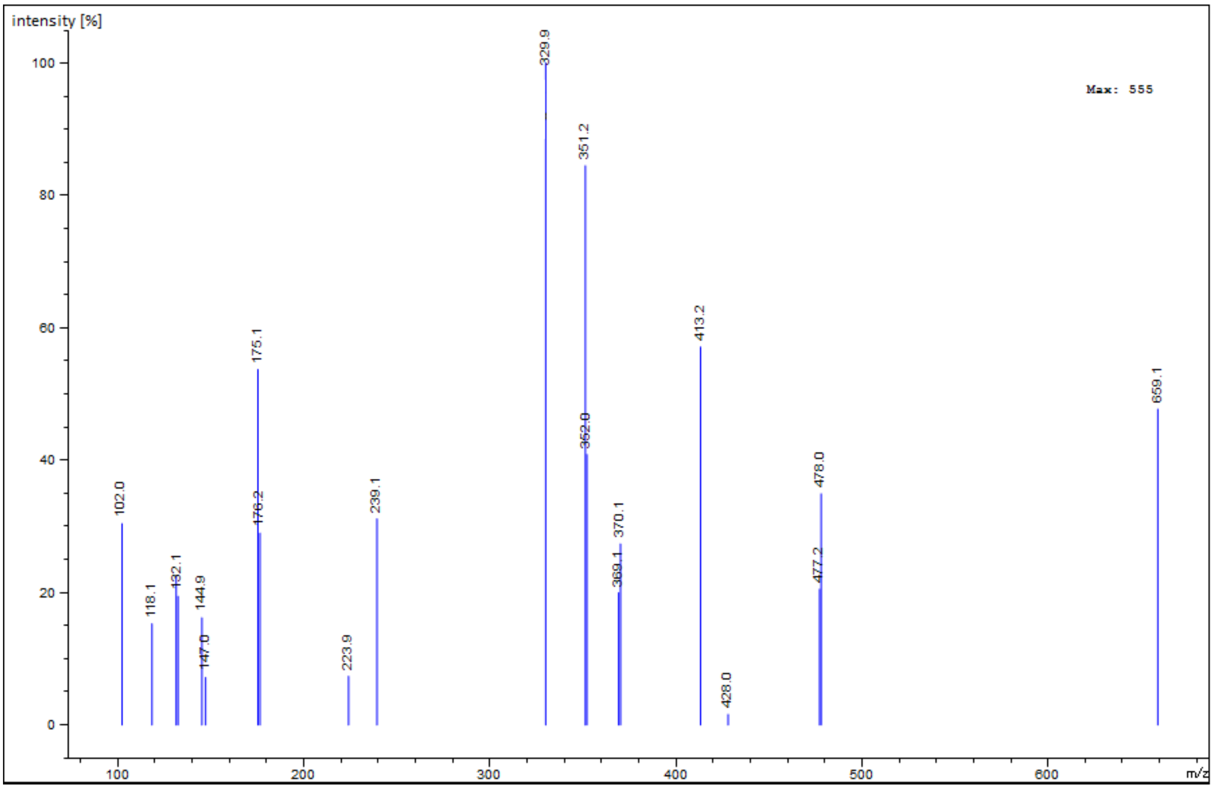

Figure S21. MS spectrum of product 6 (cGAS catalyzed reaction) ([M+H$^+$]=659.1).

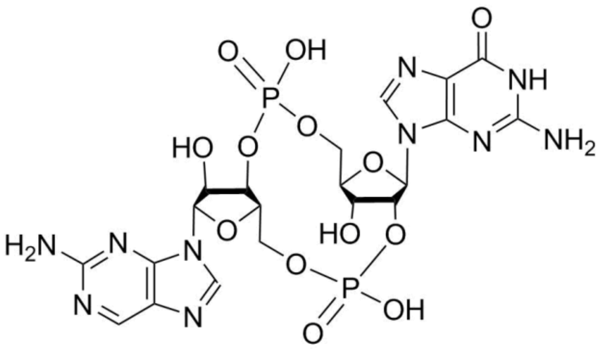

Figure S22. Chemical structure of product 7.

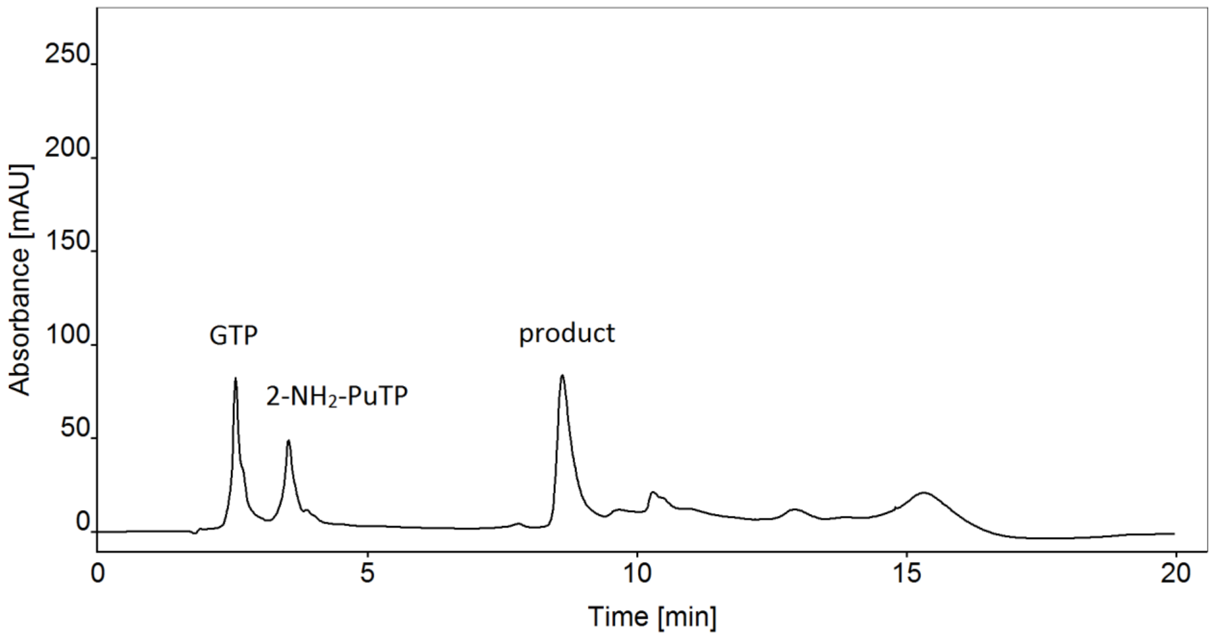

Figure S23. Chromatogram of 2-NH_2-PuTP, GTP and product 7 (cGAS catalyzed reaction).

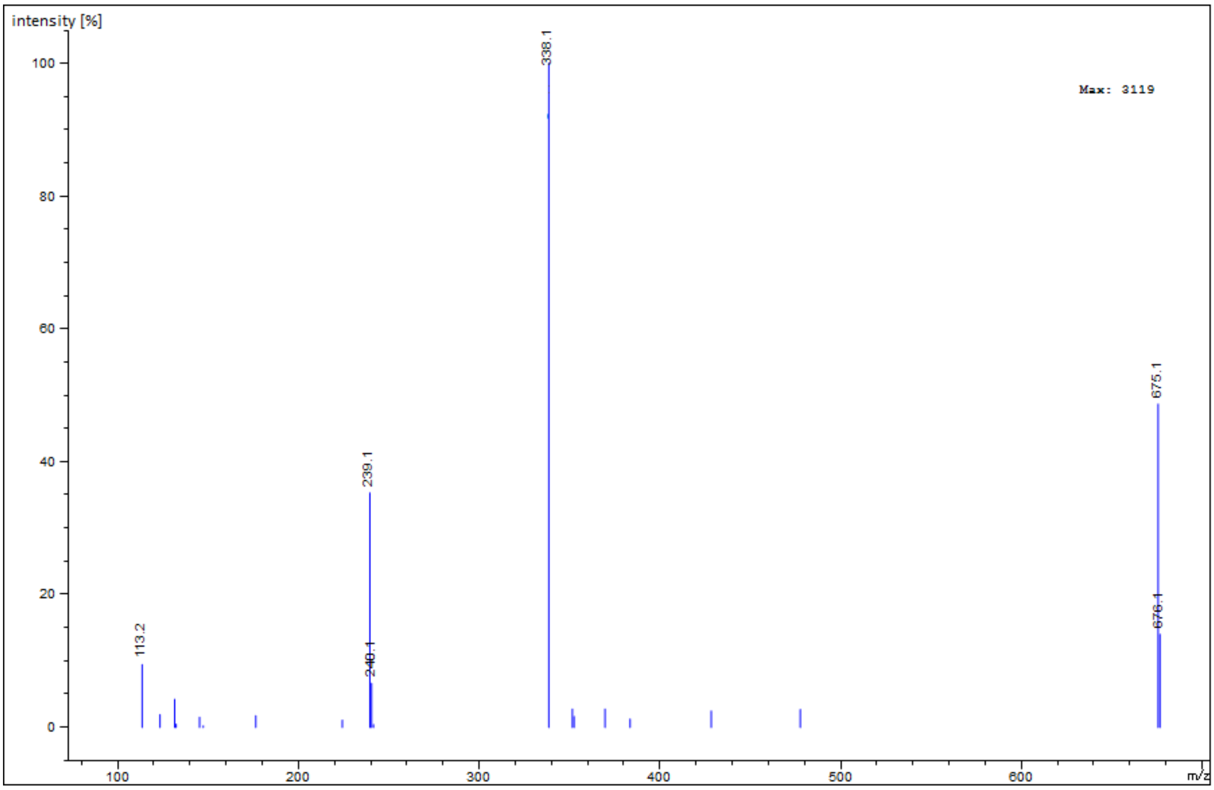

Figure S24. MS spectrum of product 7 (cGAS catalyzed reaction) ([M+H$^+$]=675.1).

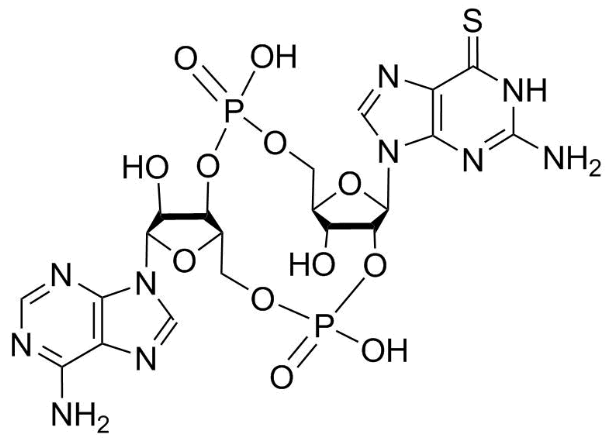

Figure S25. Chemical structure of product 8.

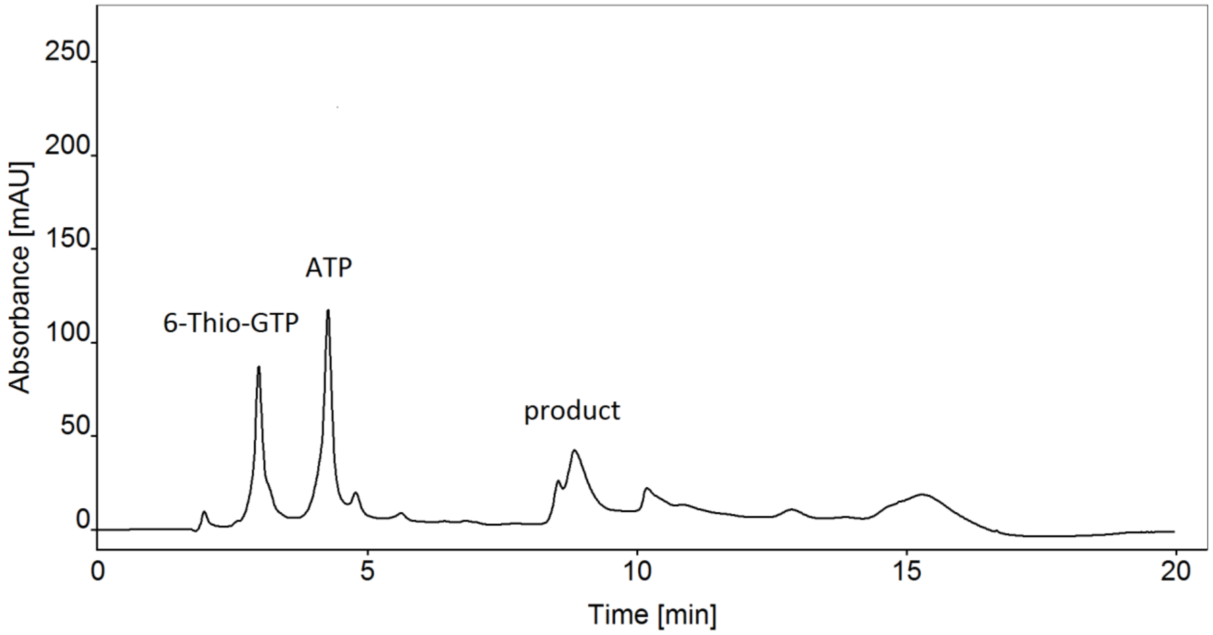

Figure S26. Chromatogram of 6-S-GTP, ATP and product 8 (cGAS catalyzed reaction).

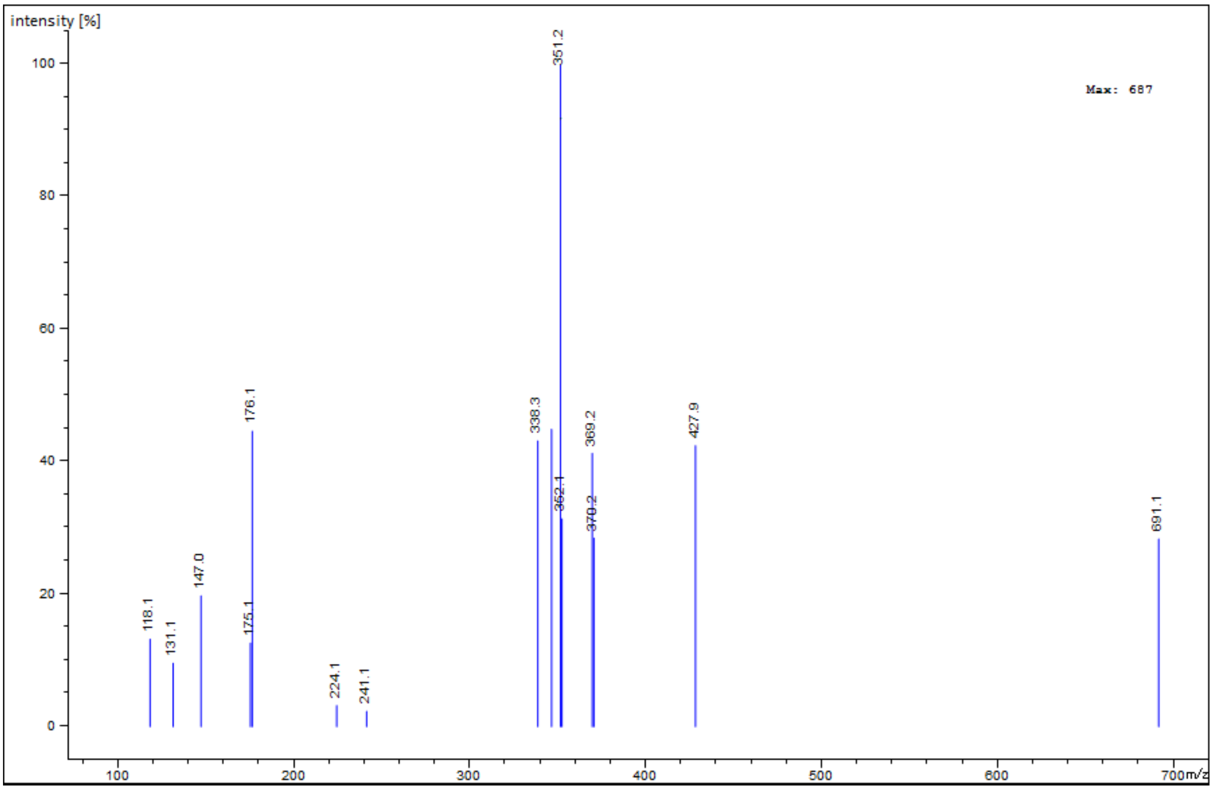

Figure S27. MS spectrum of product 8 (cGAS catalyzed reaction) ([M+H$^+$]=691.1).

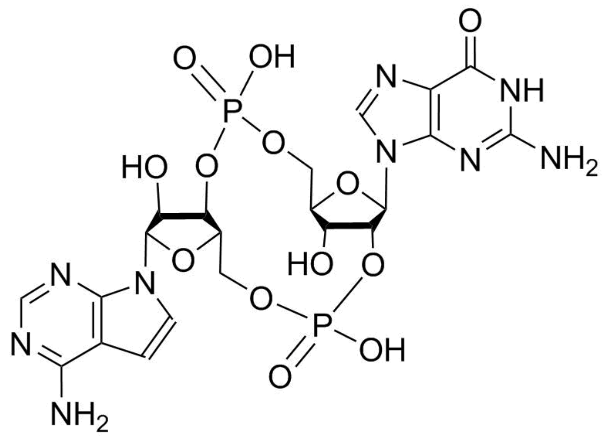

Figure S28. Chemical structure of product 9.

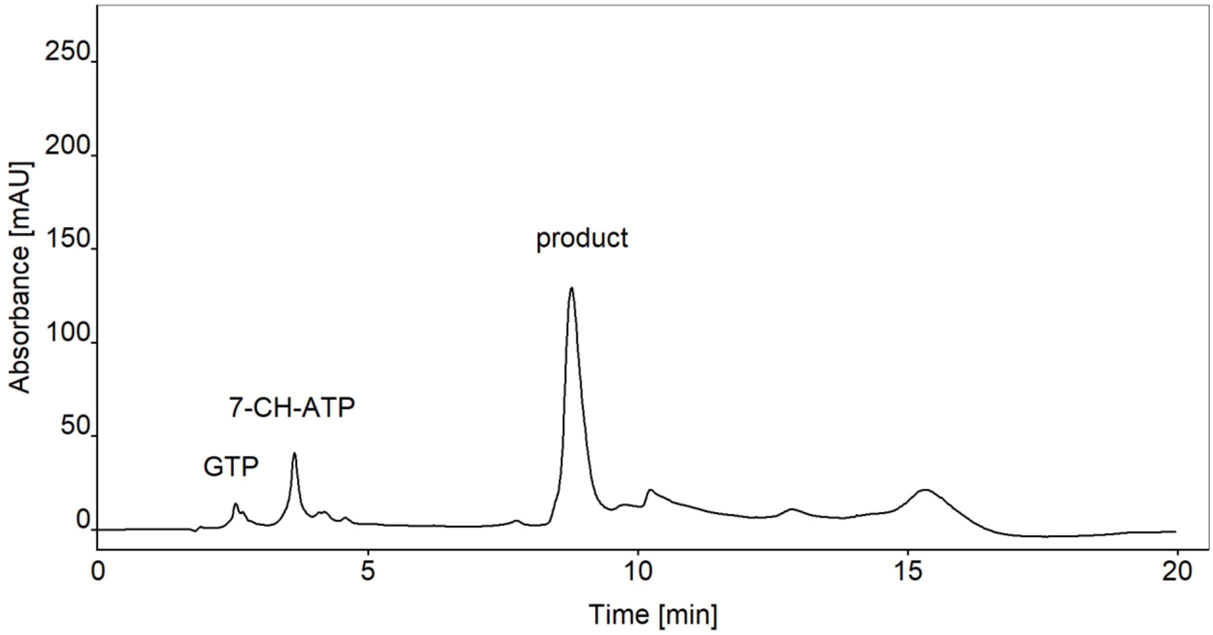

Figure S29. Chromatogram of 7-CH-GTP, ATP and product 9 (cGAS catalyzed reaction).

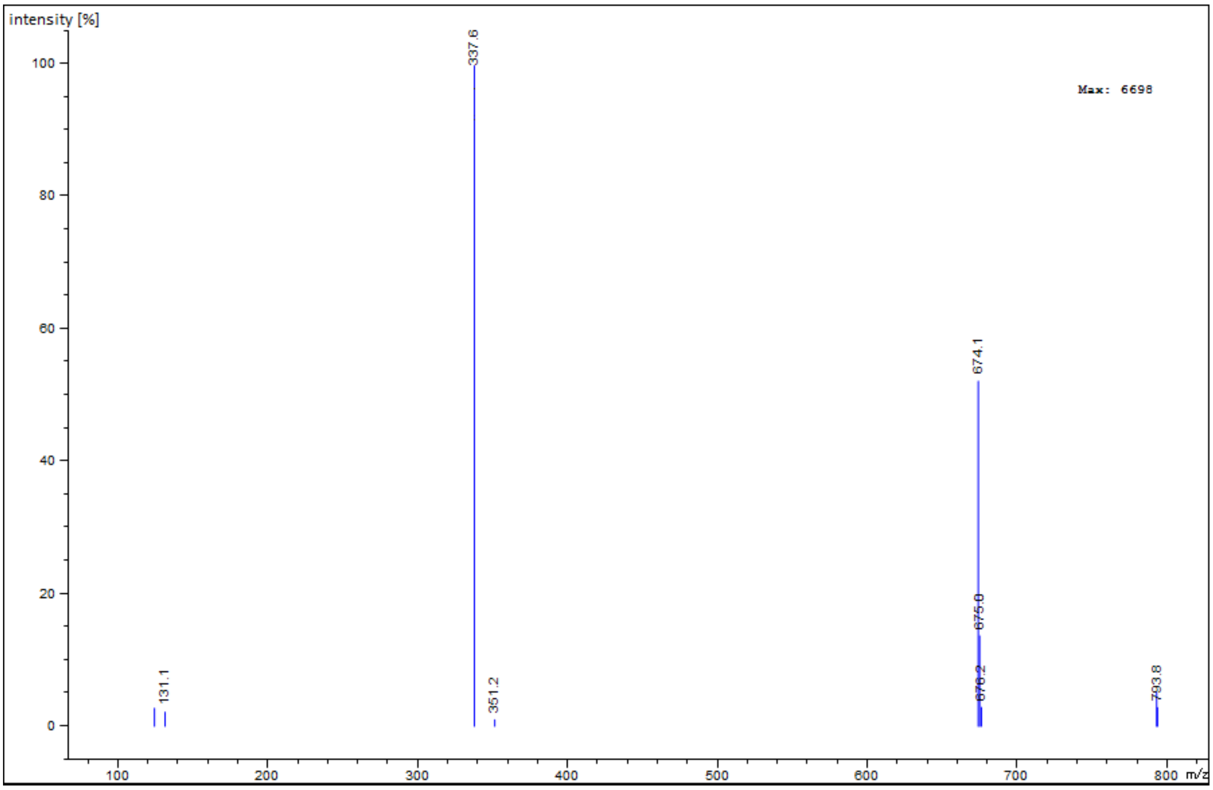

Figure S30. MS spectrum of product 9 (cGAS catalyzed reaction) ([M+H$^+$]=674.1).

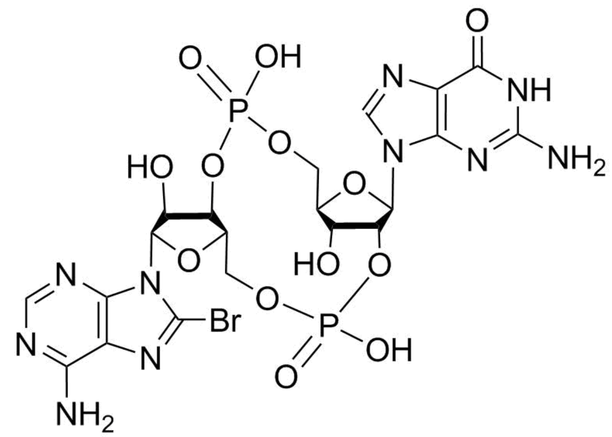

Figure S31. Chemical structure of product 10.

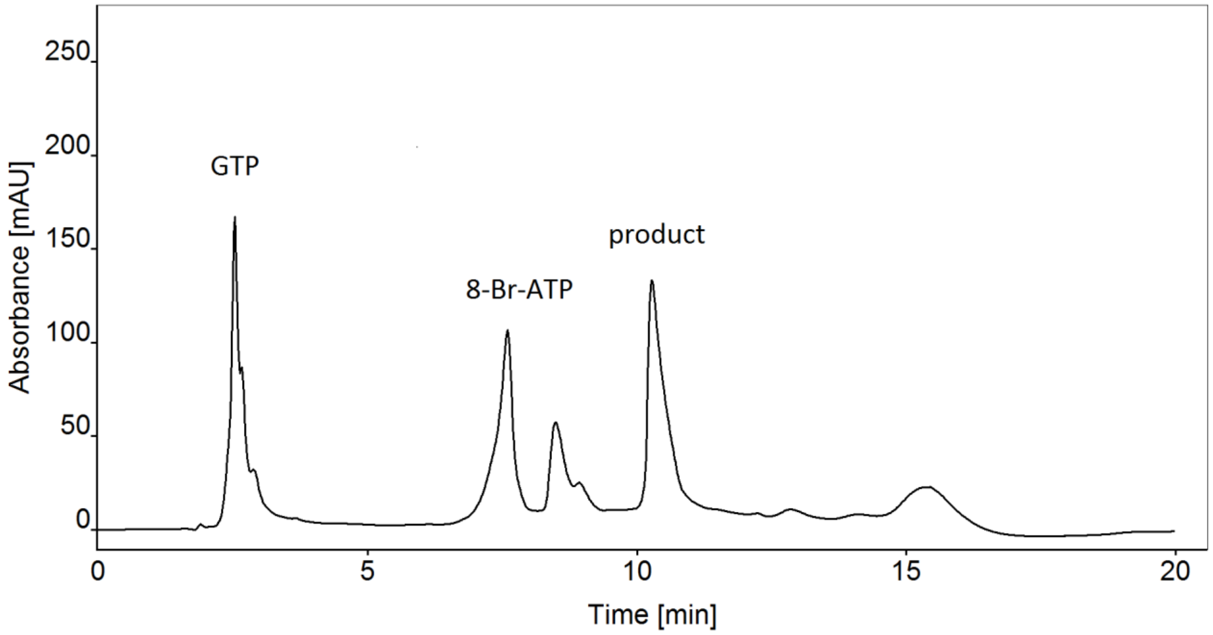

Figure S32. Chromatogram of GTP, 8-Br-ATP and product 10 (cGAS catalyzed reaction).

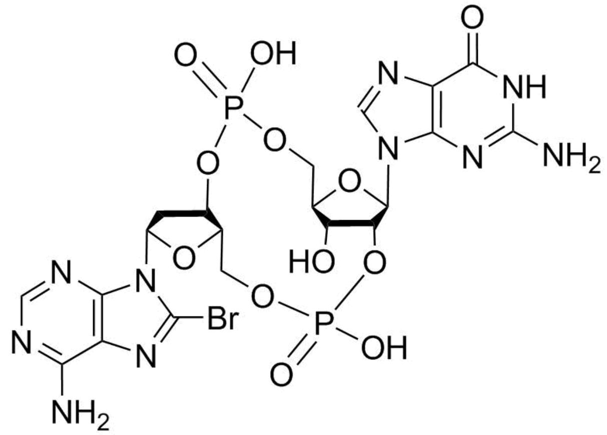

Figure 33 Chemical structure of product 11.

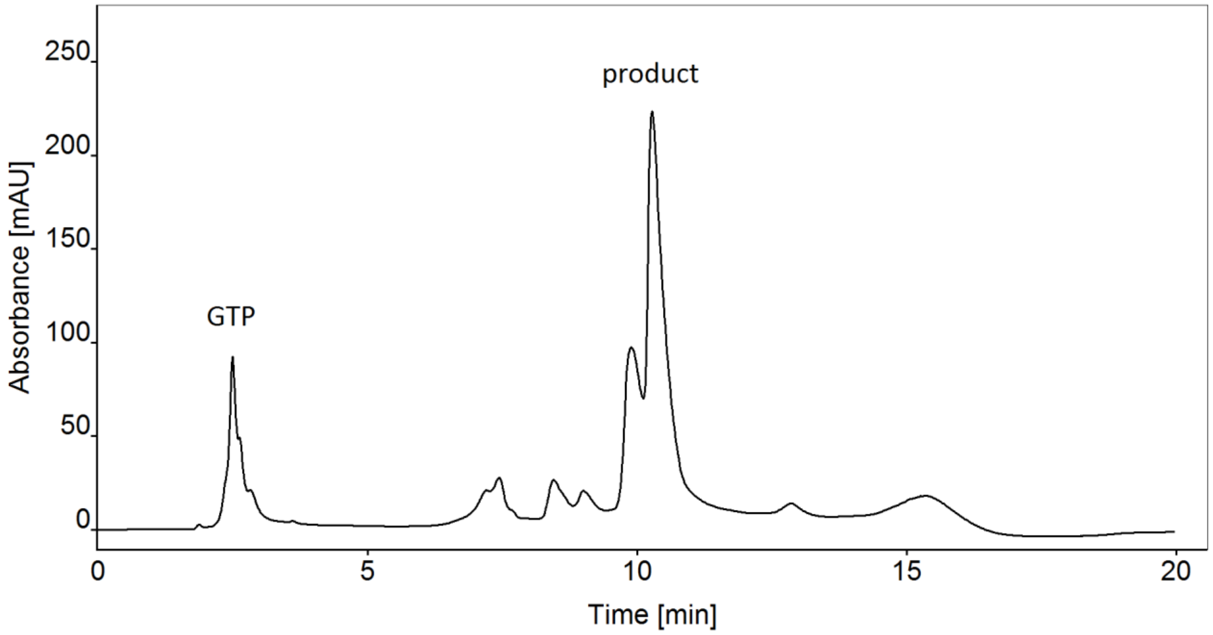

Figure S34. Chromatogram of GTP and product 11 (cGAS catalyzed reaction).

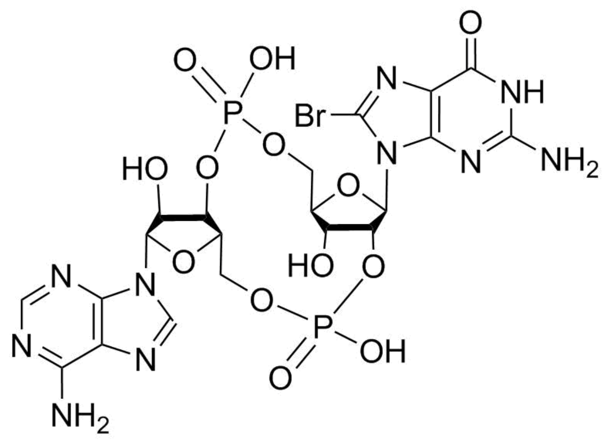

Figure S35. Chemical structure of product 12.

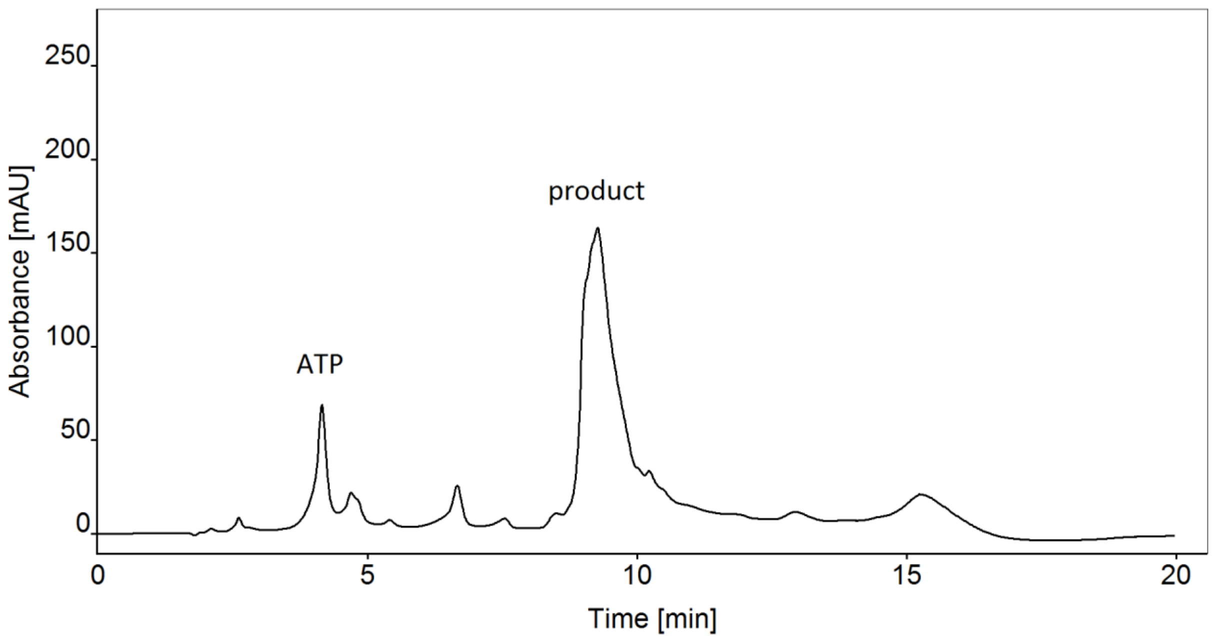

Figure S36. Chromatogram of ATP and product 12 (cGAS catalyzed reaction).

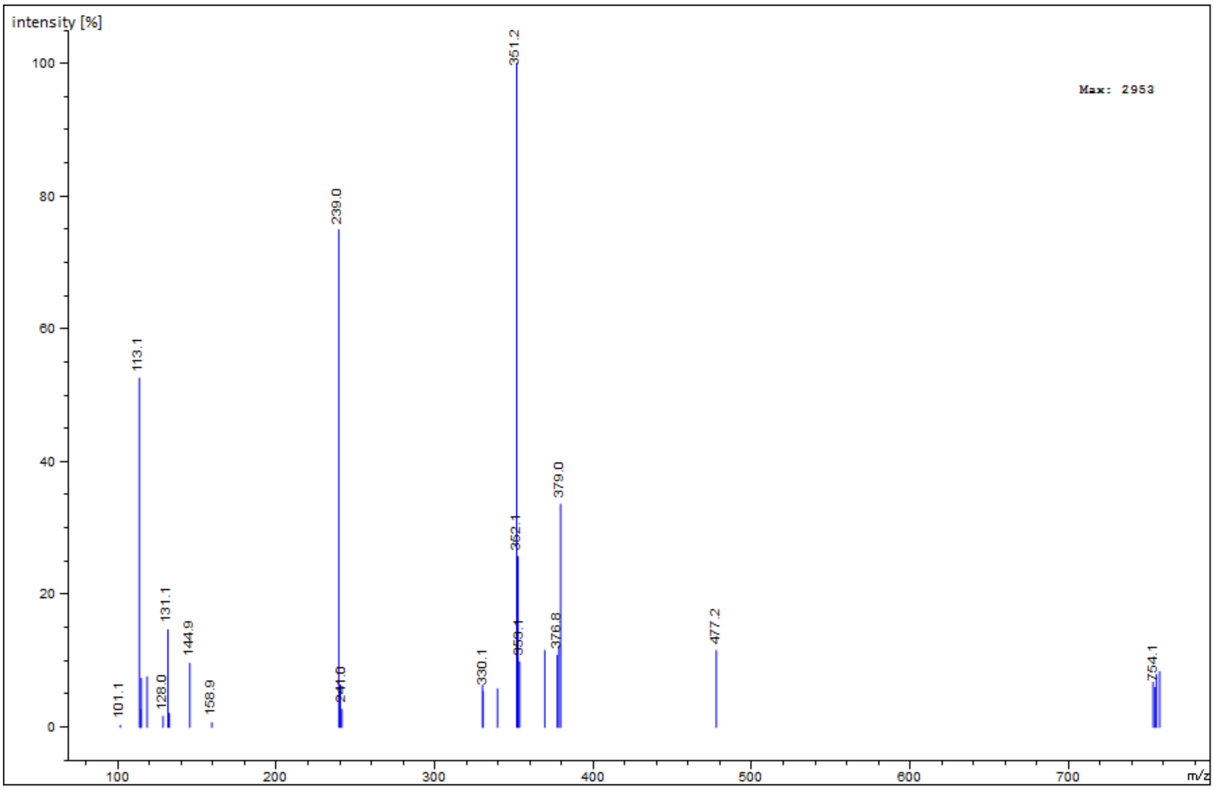

Figure S37. MS spectrum of product 12 (cGAS catalyzed reaction) ([M+H$^+$]=754.1).

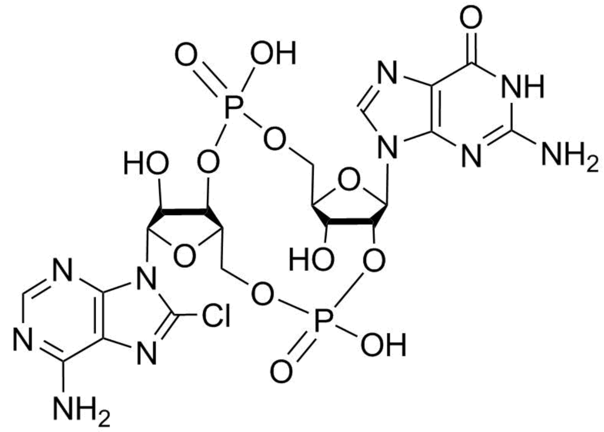

Figure S38. Chemical structure of product 13.

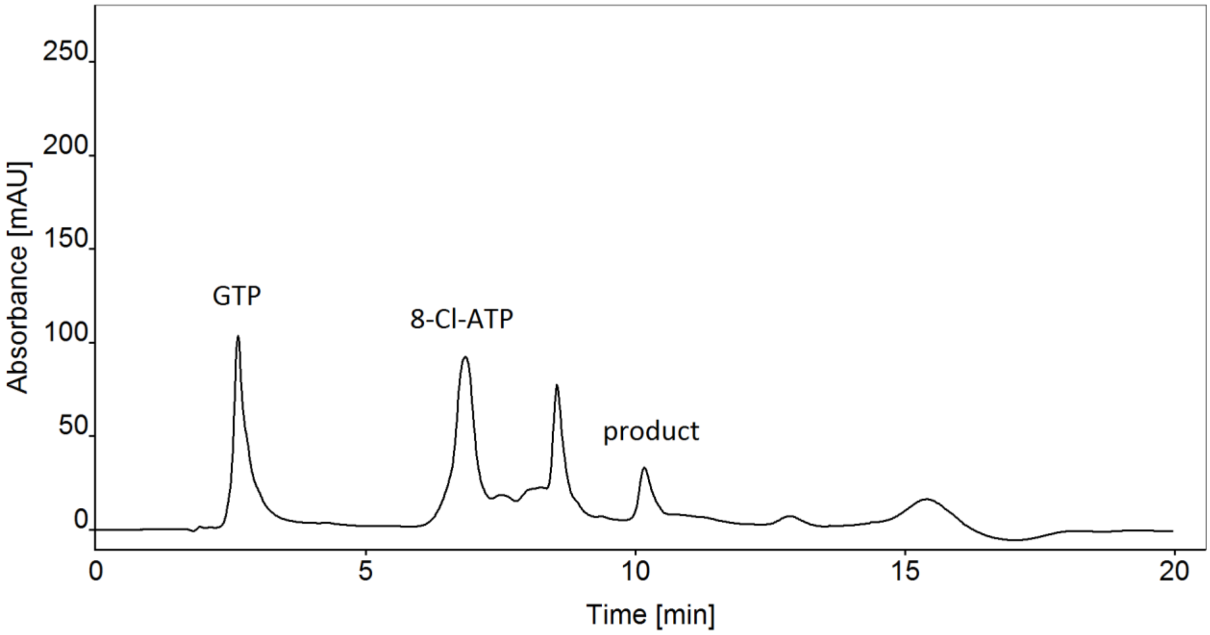

Figure S39. Chromatogram of GTP, 8-Cl-ATP and product 13 (cGAS catalyzed reaction).

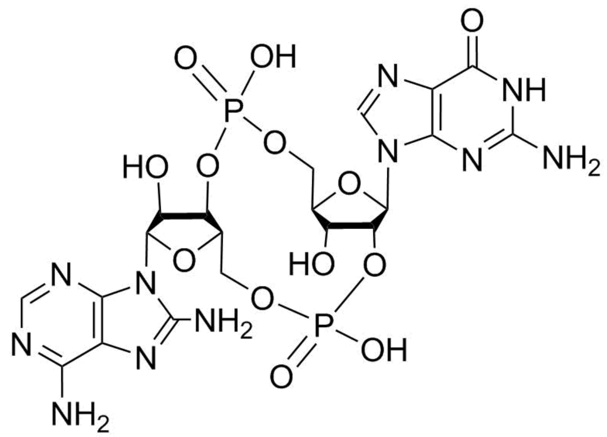

Figure S40. Chemical structure of product 14.

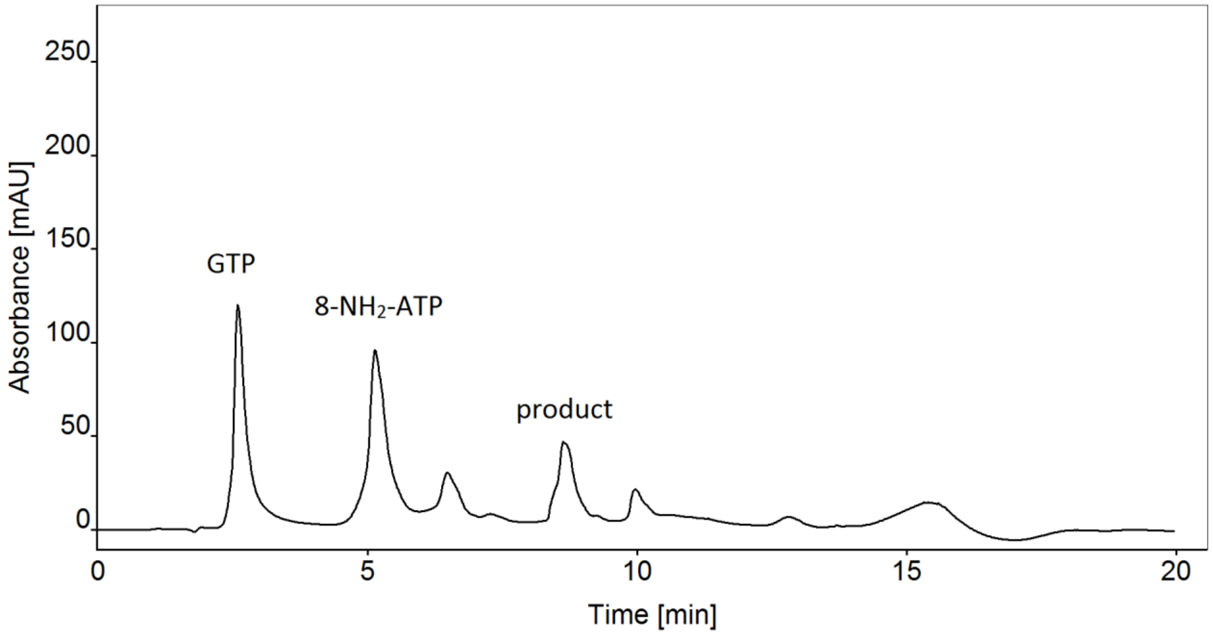

Figure S41. Chromatogram of GTP, 8-NH_2-ATP and product 14 (cGAS catalyzed reaction).

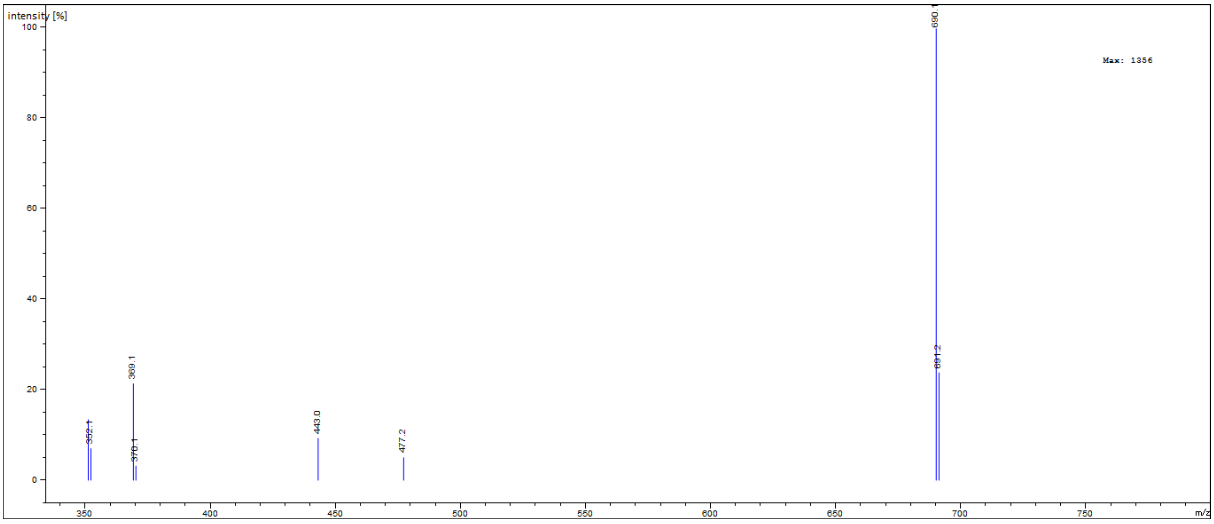

Figure S42. MS spectrum of product 14 (cGAS catalyzed reaction) ([$M+H^+$]=690.1).

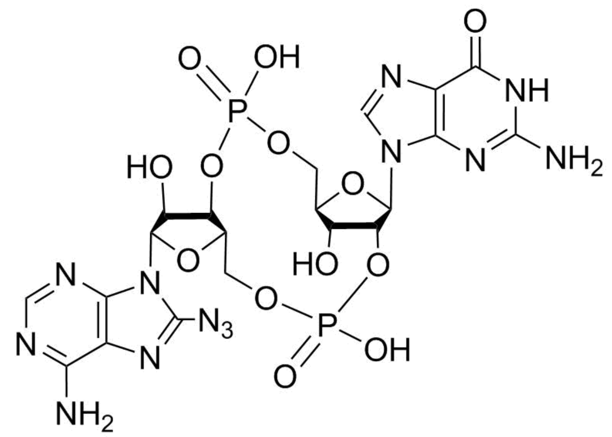

Figure S43. Chemical structure of product 15.

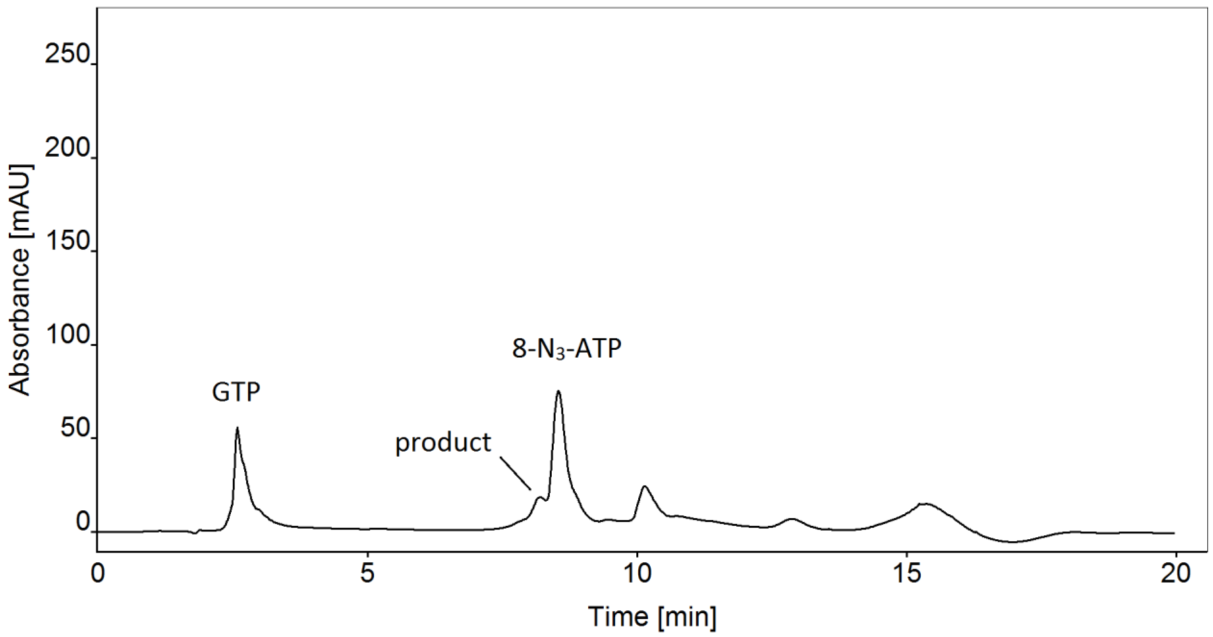

Figure S44. Chromatogram of GTP, 8-N_3-ATP and product 15 (cGAS catalyzed reaction).

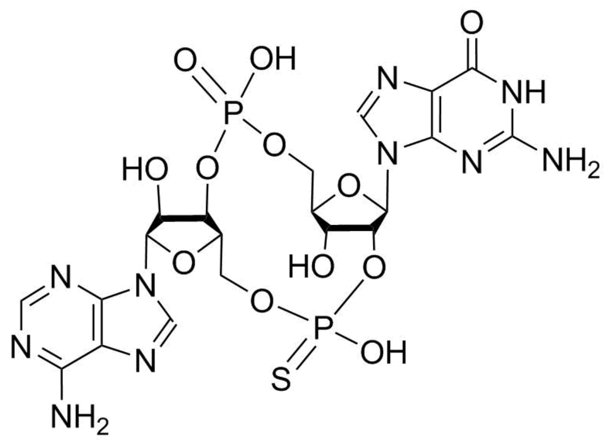

Figure S45. Chemical structure of product 16.

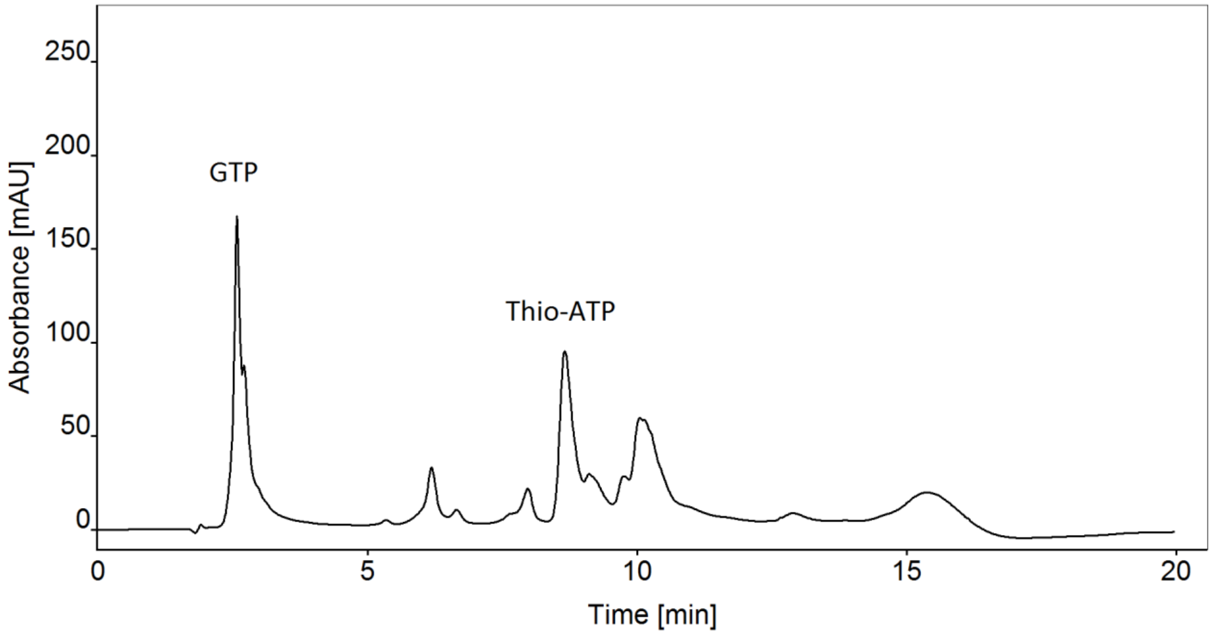

Figure S46. Chromatogram of GTP and ATP-α-S. Product 16 was not detected (cGAS catalyzed reaction).

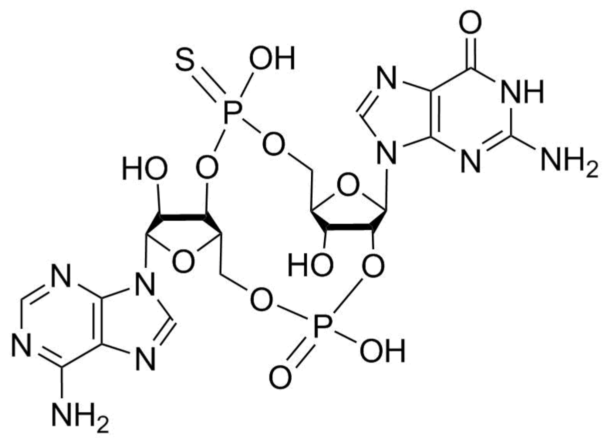

Figure S47. Chemical structure of product 17.

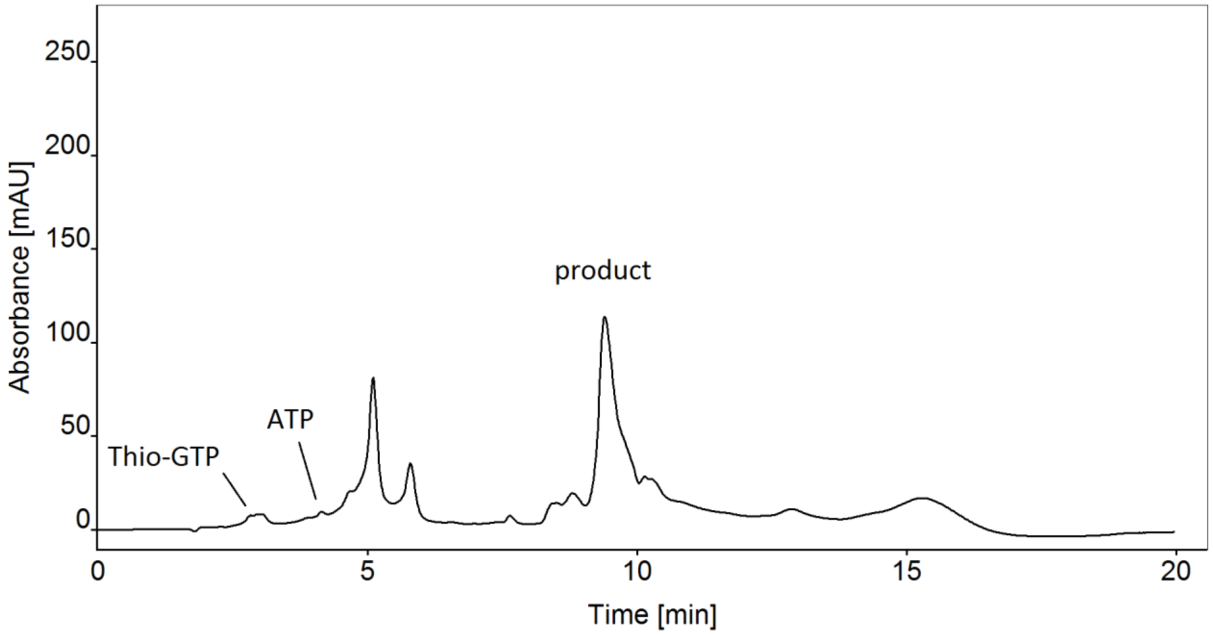

Figure S48. Chromatogram of GTP-α-S, ATP and product 17 (cGAS catalyzed reaction).

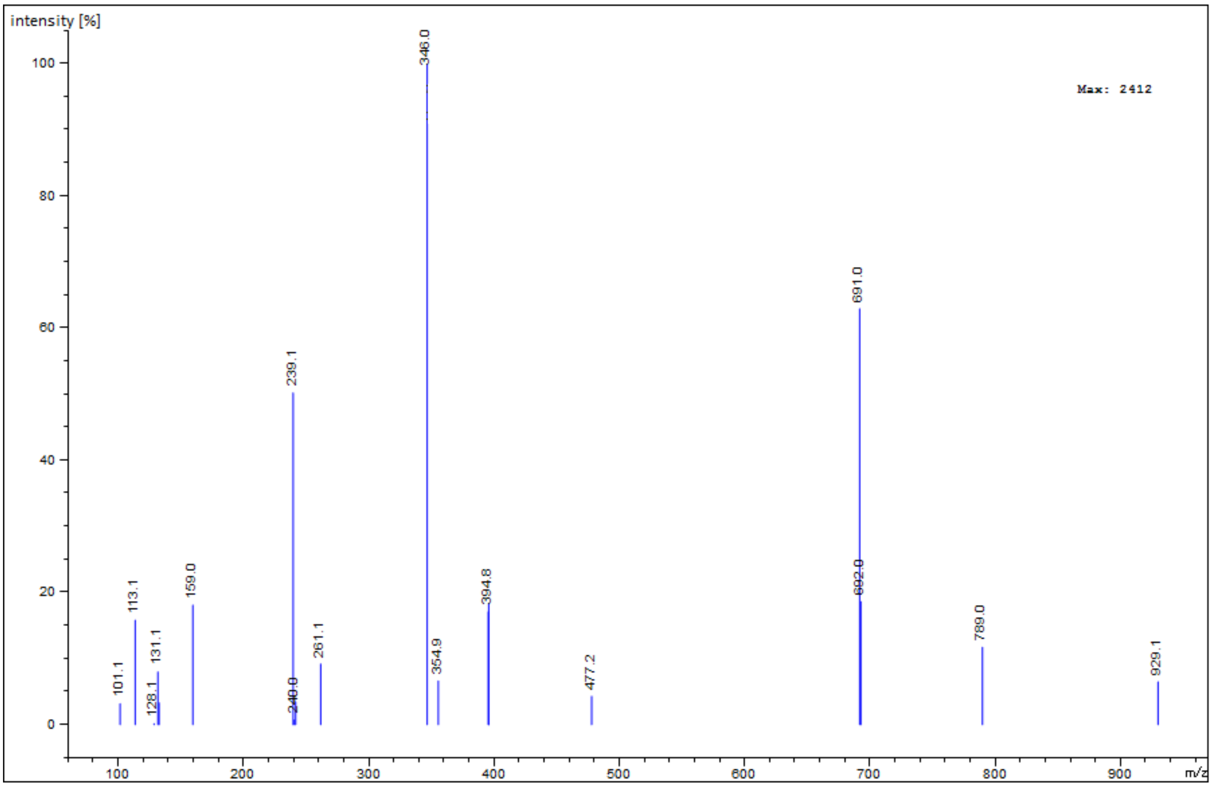

Figure S49. MS spectrum of product 17 (cGAS catalyzed reaction) ([M+H$^+$]=691.0).

NMR spectra

1D and 2D NMR spectra were recorded on a Bruker AV 600 Avance III HD system with D_2O as solvent and 25 µL methanol-d4 as internal standard. The solvent signals were referenced to δ_H 3.31 ppm and δ_C 49.0 ppm. ^1H, ^{13}C, HSQC and HMBC spectra of cyclic GMP-8-NH_2-AMP are shown. ^1H, HSQC and HMBC spectra of cyclic GMP-2'-F-AMP are shown. The concentrations of cyclic GMP-2'-F-AMP were too low to record a ^{13}C spectrum.

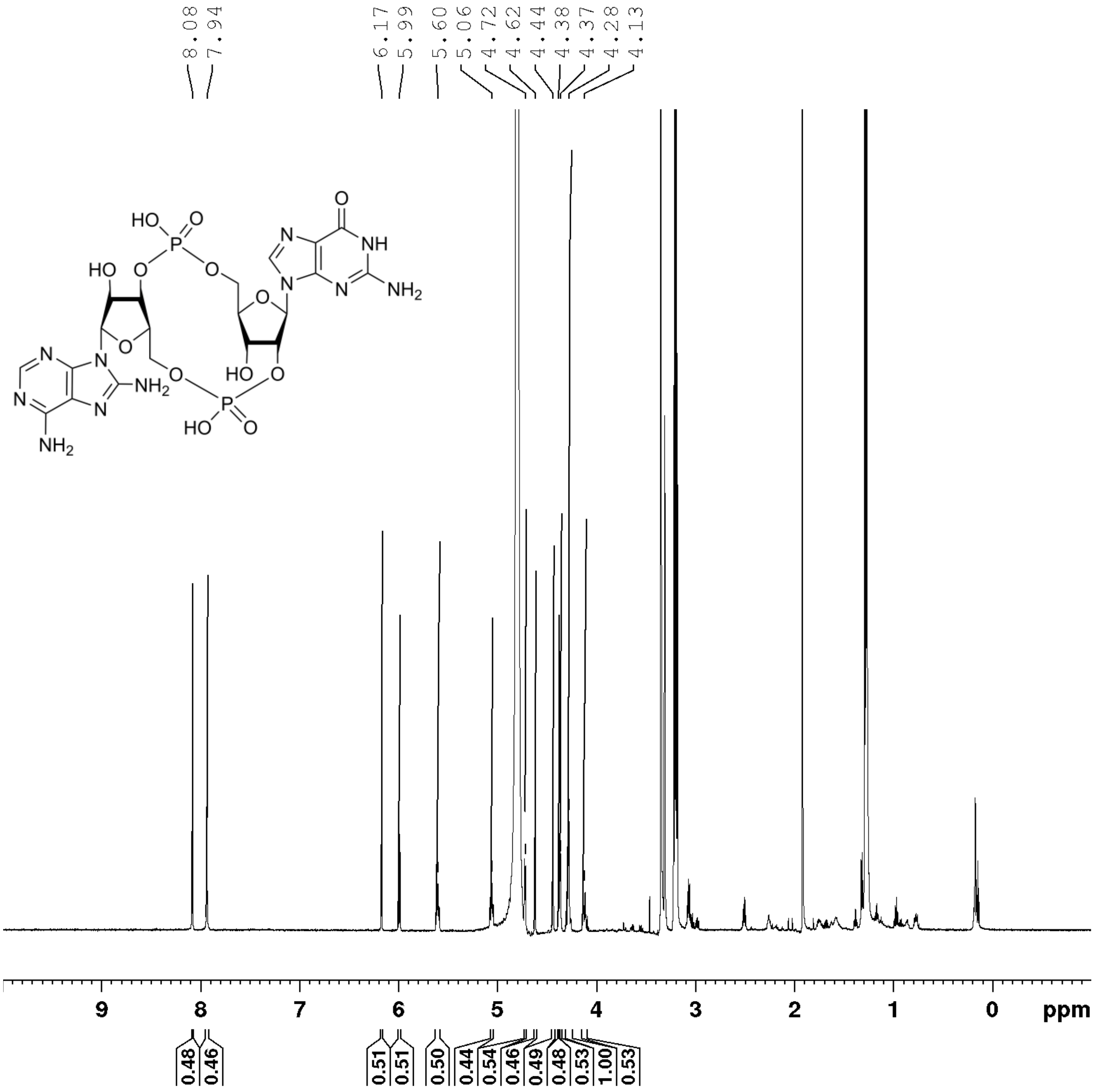

Figure S50. ^1H spectrum (600 MHz, D_2O/d_4-MeOH) of cyclic GMP-8-NH_2-AMP. Solvent and TEAA-salt impurities can be detected at δ=4.40, 3.35, 3.31, 3.20, 1.90, 1.28.

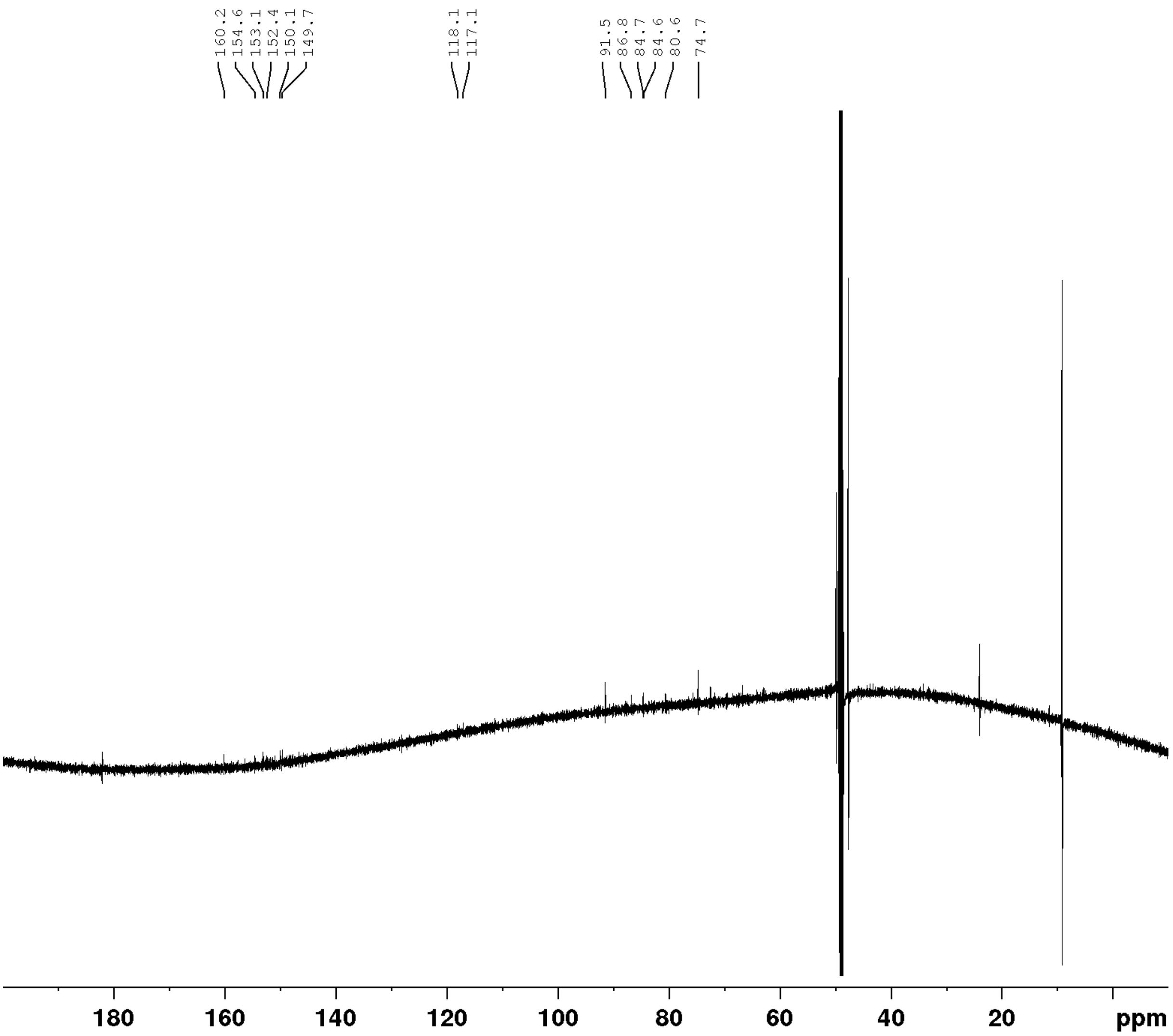

Figure S51. ^{13}C spectrum (600 MHz, D_2O/d_4-MeOH) of cyclic GMP-8-NH_2-AMP.

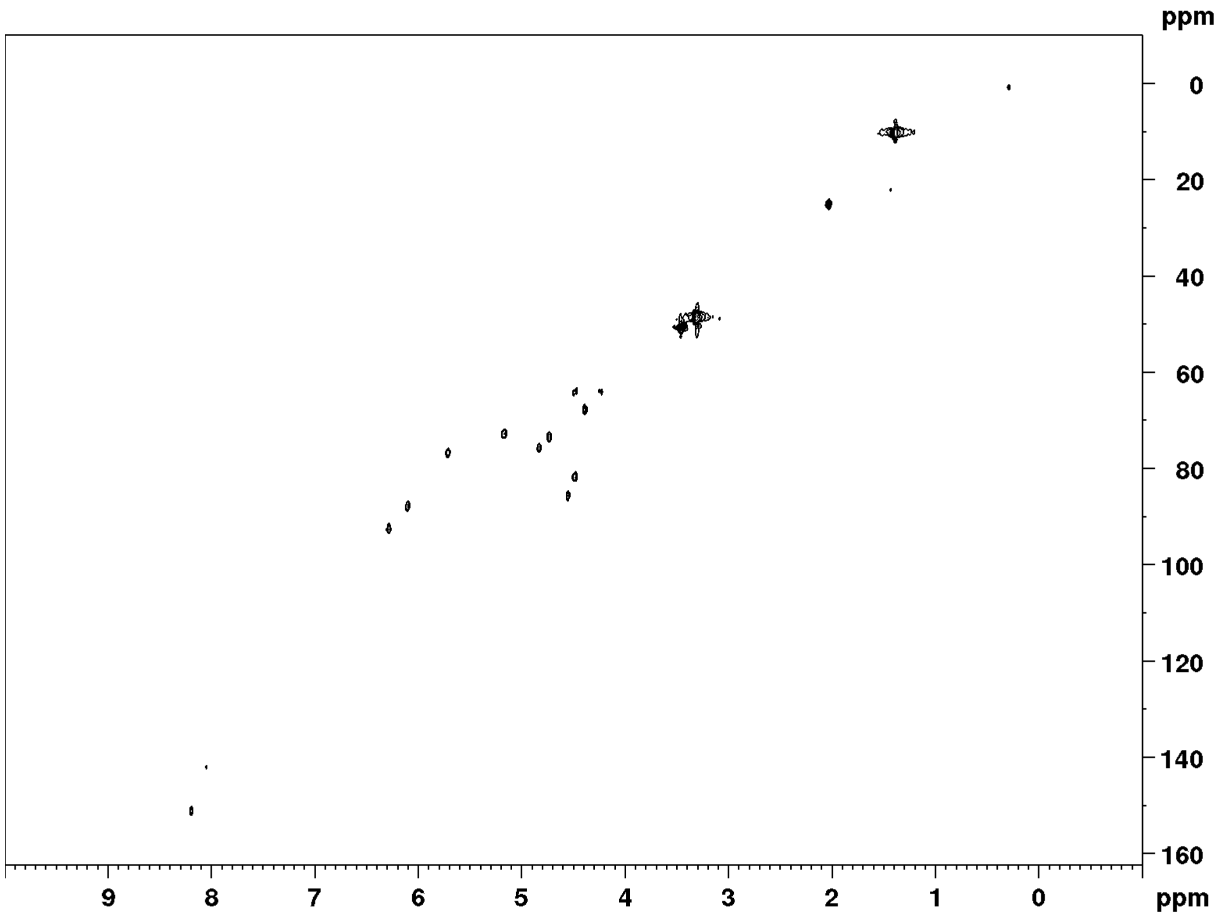

Figure S52. HSQC spectrum (600 MHz, D_2O/d_4-MeOH) of cyclic GMP-8-NH_2-AMP.

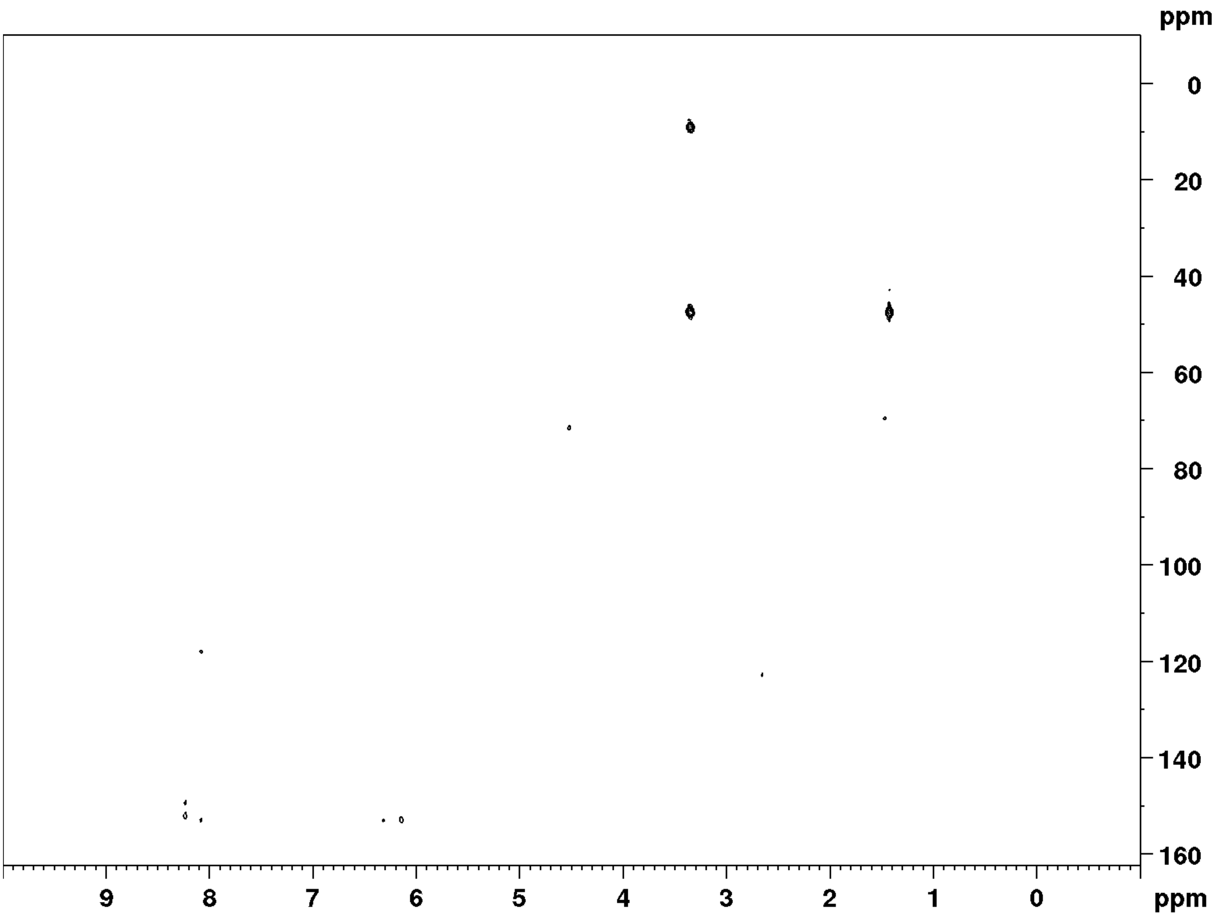

Figure S53. HMBC spectrum (600 MHz, D_2O/d_4-MeOH) of cyclic GMP-8-NH_2-AMP.

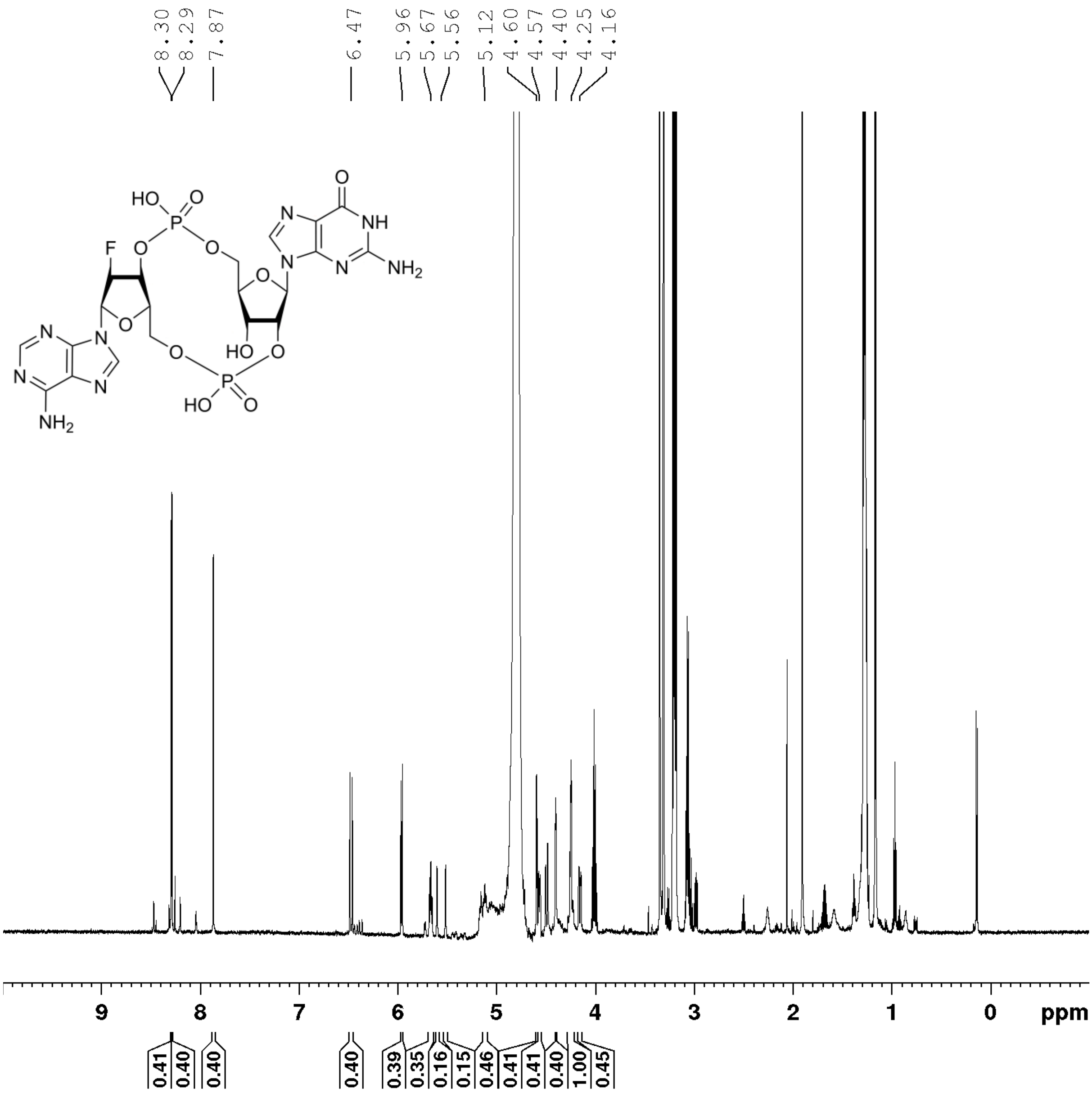

Figure S54. ^{1}H spectrum (600 MHz, D_2O/d_4-MeOH) of cyclic GMP-2'-F-AMP. Solvent and TEAA-salt impurities can be detected at δ=4.40, 3.35, 3.31, 3.20, 1.90, 1.28

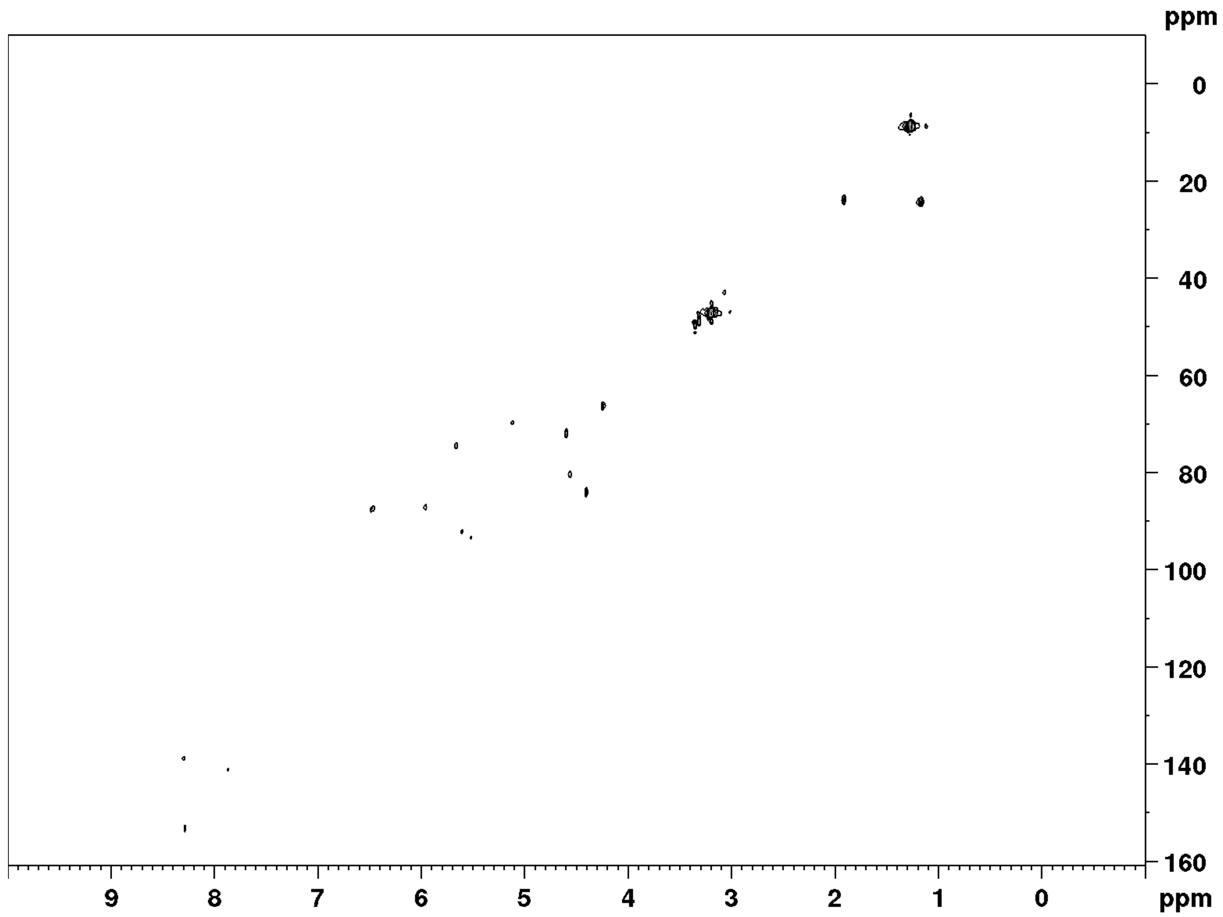

Figure S55. HSQC spectrum (600 MHz, D_2O/d_4-MeOH) of cyclic GMP-2'-F-AMP.

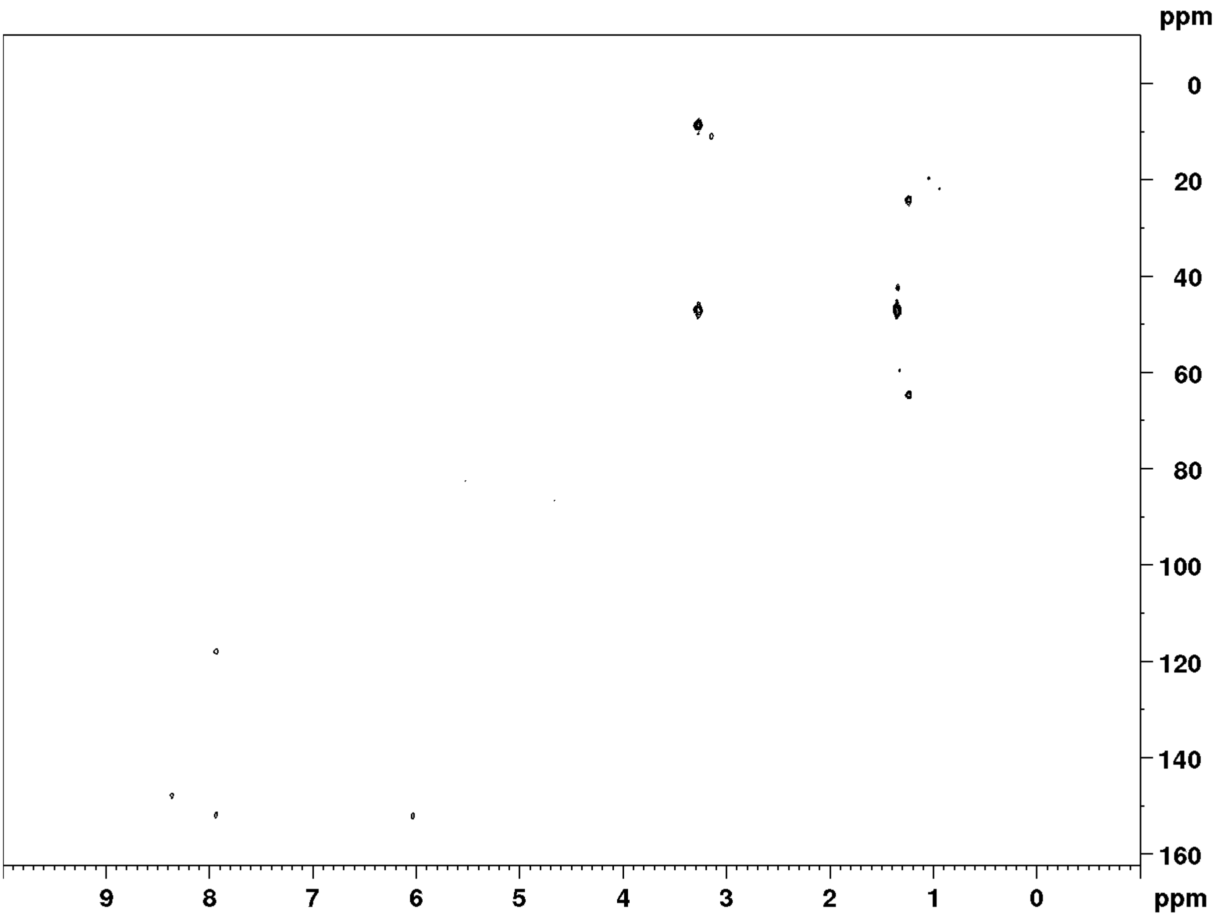

Figure S56. HMBC spectrum (600 MHz, D_2O/d_4-MeOH) of cyclic GMP-2'-F-AMP.

References

[1] J. Rolf, R. Siedentop, S. Lutz, K. Rosenthal, *Int J Mol Med* **2020**, *21*(1)
